# Pervasive RNA Regulation of Metabolism Enhances the Root Colonization Ability of Nitrogen-Fixing Symbiotic α-Rhizobia

**DOI:** 10.1128/mbio.03576-21

**Published:** 2022-02-15

**Authors:** Natalia I. García-Tomsig, Marta Robledo, George C. diCenzo, Alessio Mengoni, Vicenta Millán, Alexandra Peregrina, Alejandro Uceta, José I. Jiménez-Zurdo

**Affiliations:** a Structure, Dynamics and Function of Rhizobacterial Genomes (Grupo de Ecología Genética de la Rizosfera), Estación Experimental del Zaidín, Consejo Superior de Investigaciones Científicas (CSIC), Granada, Spain; b Department of Biology, Queen's University, Kingston, Ontario, Canada; c Department of Biology, University of Florence, Sesto Fiorentino, Italy; University of Tsukuba; University of Würzburg

**Keywords:** *Sinorhizobium meliloti*, alphaproteobacteria, noncoding RNA, riboregulation

## Abstract

The rhizosphere and rhizoplane are nutrient-rich but selective environments for the root microbiome. Here, we deciphered a posttranscriptional network regulated by the homologous *trans*-small RNAs (sRNAs) AbcR1 and AbcR2, which rewire the metabolism of the nitrogen-fixing α-rhizobium Sinorhizobium meliloti during preinfection stages of symbiosis with its legume host alfalfa. The LysR-type regulator LsrB, which transduces the cell redox state, is indispensable for AbcR1 expression in actively dividing bacteria, whereas the stress-induced transcription of AbcR2 depends on the alternative σ factor RpoH1. MS2 affinity purification coupled with RNA sequencing unveiled exceptionally large and overlapping AbcR1/2 mRNA interactomes, jointly representing ⁓6% of the S. meliloti protein-coding genes. Most mRNAs encode transport/metabolic proteins whose translation is silenced by base pairing to two distinct anti-Shine Dalgarno motifs that function independently in both sRNAs. A metabolic model-aided analysis of the targetomes predicted changes in AbcR1/2 expression driven by shifts in carbon/nitrogen sources, which were confirmed experimentally. Low AbcR1/2 levels in some defined media anticipated overexpression growth phenotypes linked to the silencing of specific mRNAs. As a proof of principle, we confirmed AbcR1/2-mediated downregulation of the l-amino acid AapQ permease. AbcR1/2 interactomes are well represented in rhizosphere-related S. meliloti transcriptomic signatures. Remarkably, a lack of AbcR1 specifically compromised the ability of S. meliloti to colonize the root rhizoplane. The AbcR1 regulon likely ranks the utilization of available substrates to optimize metabolism, thus conferring on S. meliloti an advantage for efficient rhizosphere/rhizoplane colonization. AbcR1 regulation is predicted to be conserved in related α-rhizobia, which opens unprecedented possibilities for engineering highly competitive biofertilizers.

## INTRODUCTION

Nitrogen-fixing root nodule symbioses established between some soil-dwelling alpha- or betaproteobacteria, known as rhizobia, and legume plants are pillars of agro-environmental sustainability (1). These mutualistic species-specific plant-microbe interactions rely on a strict metabolic cooperation between the partners. Rhizobia must cope with drastic shifts in nutrient availability during colonization of soil, rhizosphere, and root cells (2, 3). Bulk soil is a heterogeneous oligotrophic environment commonly exposed to abiotic stress. Plant-derived nutrients fuel the metabolism of rhizospheric and invading rhizobia, as well as symbiotic nitrogen fixation by morphologically differentiated bacteroids within nodules. Thus, the success of a symbiosis greatly depends on the ability of rhizobia to accurately adapt their metabolism to the nutritionally complex soil and plant environments. The metabolic versatility of rhizobia is supported by large and multipartite genomes, with a generous genetic endowment arranged in complex networks devoted to nutrient uptake and catabolism (4, 5). To date, regulation of these metabolic pathways has been almost exclusively attributed to proteins involved in differential transcription (i.e., transcription factors and alternative RNA polymerase holoenzymes) or specific posttranslational modifications, but the underlying posttranscriptional mechanisms are largely unknown (6–9). However, since the massive discovery of small noncoding RNAs (sRNAs) in prokaryotes, RNA-mediated posttranscriptional control of metabolism has been regarded as a ubiquitous level of regulation, contributing greatly to bacterial fitness in fluctuating environments (10).

The best-characterized noncoding transcriptome in rhizobia is that of the alphaproteobacterium Sinorhizobium meliloti, the symbiont of alfalfa (Medicago sativa L.) and other medic legumes (11–13). Transcriptome sequencing (RNA-seq) surveys have unveiled a large and heterogeneous population of noncoding RNAs expressed by S. meliloti, but only a few so-called *trans*-sRNAs involved in regulating quorum sensing, the cell cycle, and metabolism have been further characterized (14–20). *trans*-acting sRNAs constitute the most studied class of riboregulators in bacteria. It includes transcripts mostly encoded within intergenic regions (IGRs) that typically influence the translation and/or half-life of multiple *trans*-encoded mRNAs upon protein-assisted short and imperfect base pairing (21). Computational predictions and preliminary experimental data suggest that at least three of the characterized S. meliloti *trans*-sRNAs, namely, NfeR1, AbcR1, and AbcR2, regulate nutrient uptake by inhibiting the translation of mRNAs encoding components of ABC transporters (14, 15, 22).

NfeR1 (nodule formation efficiency RNA 1) is a 115-nucleotide (nt) stress-induced sRNA required for osmoadaptation and nodule development (14). The regulatory ability of NfeR1 depends on three identical anti-Shine-Dalgarno (aSD) motifs that contribute redundantly to translation inhibition. Only two mRNAs have been experimentally validated as NfeR1 targets, *SMc03121* and *SMb20442*, both of which encode periplasmic substrate-binding proteins of yet-uncharacterized transporters. AbcR1 and AbcR2 (ATP-binding cassette regulators 1 and 2) are 121-nt- and 114-nt-long homologous sRNAs tandemly carried in the IGR flanked by the genes *SMc01226* and *lsrB*, which encode an ArsR-type and the LsrB (LysR-type symbiotic regulator B) transcriptional regulators, respectively (15). Unlike NfeR1, AbcR1/2 interact with Hfq, the widespread bacterial chaperone that commonly acts as an RNA matchmaker in riboregulation (22). Despite their homology, these sRNAs are differentially expressed (15). AbcR1 expression is induced during exponential growth and at early root infection, while it is downregulated in nitrogen-fixing endosymbiotic bacteroids. Conversely, the highest levels of AbcR2 accumulation occur at the onset of stationary-phase growth and in response to abiotic stress, whereas it is barely detected *in planta* throughout symbiosis. Accordingly, only a lack of AbcR1 results in a growth delay in rich medium, and both AbcR1 and AbcR2 are dispensable for wild-type nodule organogenesis and nitrogen fixation in symbiosis with M. sativa plants (15).

S. meliloti AbcR1/2 are founding members of the so-called αr15 family of alphaproteobacterial sRNAs, which has representatives in most *Rhizobiaceae* and *Brucellaceae* species, including the mammal and plant pathogens Brucella abortus and Agrobacterium tumefaciens (23–25). Remarkably, AbcR1 and AbcR2 are functionally redundant for B. abortus virulence; i.e., only a double deletion prevents chronic infection of host macrophages (26, 27). In A. tumefaciens and B. abortus, AbcR1 expression is regulated by VtlR, the LysR-type transcriptional regulator (LTTR) encoded by the neighboring gene (28, 29). The AbcR1 genomic region shows high synteny across alphaproteobacteria, and therefore, the S. meliloti ortholog LsrB has been proposed as the most probable AbcR1 regulator in this bacterium. However, this hypothesis has not yet been experimentally demonstrated (30). Transcriptomic and proteomic signatures of knockout mutants have drafted the regulons of B. abortus and A. tumefaciens AbcR1/2 (26, 27, 31). Although such profiling does not discriminate between directly and indirectly regulated mRNAs, differentially expressed transcripts were functionally enriched in nutrient uptake and virulence factors. Further experimental validation demonstrated that subsets of these target mRNA candidates are regulated through either of the two aSD motifs that are identifiable as single-stranded regions in the predicted secondary structures of αr15 sRNAs (27, 31). Similarly, genetic reporter assays have confirmed *livK*, *prbA*, and *SMa0495*, all encoding the periplasmic component of ABC transporters for amino acid uptake, as targets of S. meliloti AbcR1/2 (15, 22). These mRNAs are most probably regulated by base pairing of the aSD motifs to the ribosome binding site (RBS) and flanking nucleotides, but these interactions have not yet been genetically dissected.

Here, we explored the regulation of the S. meliloti AbcR1/2 sRNAs and uncovered their mRNA interactomes using MAPS (MS2 affinity purification coupled with RNA sequencing) (32, 33). Our data show that LsrB and the alternative σ factor RpoH1 are responsible for the differential transcription of AbcR1 and AbcR2, respectively. In turn, these sRNAs use their aSD motifs to downregulate the translation of large and overlapping arrays of mRNAs encoding transport proteins and metabolic enzymes. Further, we show that AbcR1-mediated posttranscriptional fine-tuning of metabolism enhances the ability of S. meliloti to colonize the root rhizoplane, a biotechnologically relevant symbiotic trait.

## RESULTS

### Regulators of AbcR1/2 transcription.

To identify putative functional motifs in the AbcR1/2 promoters (P*_abcR1_* and P*_abcR2_*), we compared the 100 nucleotides preceding the transcription start sites of these genes and their predicted homologs in several alphaproteobacteria (see Fig. S1A in the supplemental material). P*_abcR1_* alignment unveiled the −35/−10 core hexamers recognized by RpoD (σ^70^) in alphaproteobacteria (CTTGAC-N_17_-CTATAT) (11). Upstream of this σ^70^ signature, we noticed the generic LTTR motif of prokaryotic promoters (T-N_11_-A), which occurs in tandem in most sequences. Remarkably, a more defined motif perfectly matching the proposed LsrB-binding consensus GCAT-N_3_-TG-N_3_-T in B. abortus and A. tumefaciens was also evident between the −61 and −49 positions in P*_abcR1_*. Comparison of the P*_abcR2_* sequences revealed a −35/−10 box closely matching the S. meliloti RpoH1 (σ^H1^) consensus sequence (CTTGAA-N_16_-CCTATAT) but failed to detect additional conserved motifs (34).

10.1128/mbio.03576-21.2 FIG S1 Regulation of AbcR1/2 expression. (A) Multiple-sequence alignment of the promoters of AbcR1/2 homologs in alphaproteobacteria. Consensus of conserved motifs are indicated below the alignments. *Sm*; Sinorhizobium meliloti Sm1021; *Smed*, *S. medicae* WSM419; *Sfr*, *S. fredii* HH103; *Rlv*, Rhizobium leguminosarum bv. viciae 3841; *Rltr*, R. leguminosarum bv. trifolii WSM2304; *Ret*, *R. etli* CIAT652; *Bab*, Brucella abortus 2308; *At*, Agrobacterium tumefaciens C58. (B) LsrB binds the *abcR1* promoter. Gel shift assays with radiolabeled P*_abcR1_* (334 bp) and P*_abcR2_* (206 bp) incubated with purified LsrB. (C) RpoH1/2 contribute to AbcR2 transcription upon an osmotic upshift. Northern blot probing with the PbAbcR2 oligonucleotide of RNA from Sm1021 and its *rpoH1, rpoH2*, and *rpoH1/2* knockout mutants cultured under the conditions indicated along the top of the panel. The 5S rRNA was probed as an RNA loading control. Download FIG S1, TIF file, 1.1 MB. Copyright © 2022 García-Tomsig et al. 2022 García-Tomsig et al. https://creativecommons.org/licenses/by/4.0/ This content is distributed under the terms of the Creative Commons Attribution 4.0 International license.

**FIG 1:**
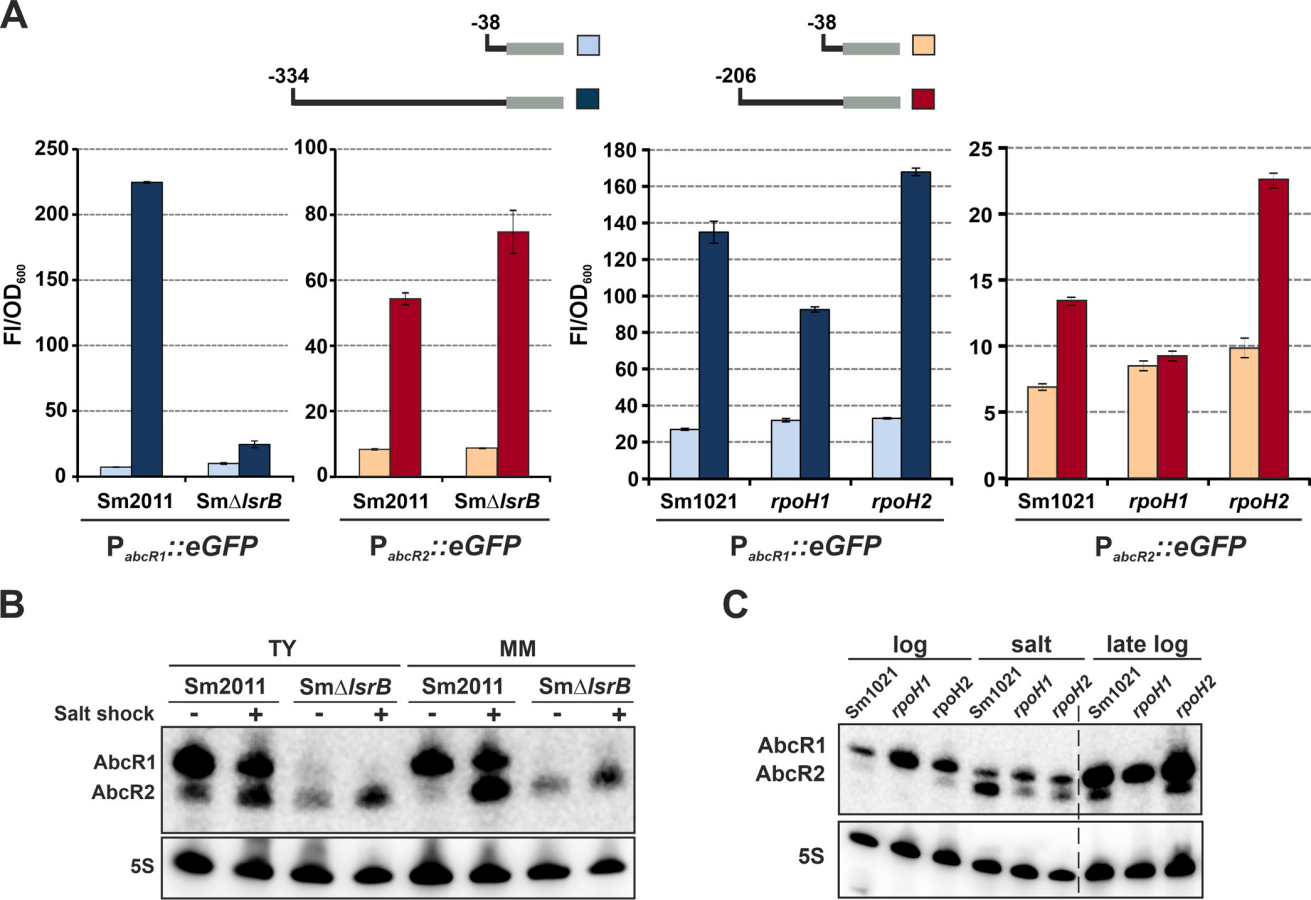
Transcriptional regulation of S. meliloti AbcR1 and AbcR2 sRNAs. (A) Fluorescence of promoter-*eGFP* fusions. Fluorescence derived from full-length and trimmed versions of P*_abcR1_* and P*_abcR2_*, as diagrammed above the bar graphs, were determined in wild-type (Sm2011 or Sm1021) and mutant *lsrB* (SmΔ*lsrB*), *rpoH1*, and *rpoH2* backgrounds. Reported values are means and standard deviations (SD) of nine fluorescence measurements normalized to the OD_600_ (Fl/OD_600_) of exponential (P*_abcR1_*)- and stationary (P*_abcR2_*)-phase cultures, i.e., three replicates of three independent cultures of each reporter strain. (B) Northern blot analysis of LsrB-dependent AbcR1/2 expression. Total RNA was obtained from Sm2011 and SmΔ*lsrB* bacteria. (C) Northern blot analysis of RpoH1/2-dependent AbcR1/2 expression. Total RNA was extracted from Sm1021 and *rpoH1 rpoH2* knockout mutants. Growth conditions are indicated on top of each panel. Membranes were probed with the PbAbcR1/2-radiolabeled oligonucleotide. The 5S rRNA was probed as an RNA loading control. Hybridizations shown are representatives of at least three biological replicates per strain and growth condition.

To experimentally test these predictions, we first transcriptionally fused full-length (P*_abcR1_*/P*_abcR2_*) and truncated (P*_abcR1–38_*/P*_abcR2–38_*) versions of both promoters to the enhanced green fluorescent protein gene (*eGFP*). All four reporter constructs were independently introduced into wild-type S. meliloti (Sm2011 and Sm1021) and *lsrB* (SmΔ*lsrB*), *rpoH1*, and *rpoH2* knockout mutant strains (Fig. 1A). Strains Sm2011 and Sm1021 (reference genome) derive from the same S. meliloti nodule isolate (SU47). Both are considered nearly isogenic (Table S1), but strikingly, we failed to generate an *lsrB* knockout in Sm1021 using both single- and double-crossover strategies, suggesting that the genomic background can affect *lsrB* mutant viability. The activities of the promoters were assessed in bacteria grown to exponential (P*_abcR1_*) or stationary (P*_abcR2_*) phase in complete tryptone-yeast (TY) medium, which induces AbcR1 or AbcR2 expression, respectively. The maximum fluorescence of the P*_abcR1_*::*eGFP* reporter fusion was detected in Sm2011, decreasing by >22-fold upon removal of the predicted LsrB-binding motif. In the absence of LsrB, the activity of P*_abcR1_* was merely double that of P*_abcR1–38_*. Conversely, the lack of LsrB did not significantly alter P*_abcR2_*’s activity. Gel shift assays further demonstrated the binding of LsrB to P*_abcR1_* (Fig. S1B). In strain Sm1021 and its *rpoH* insertion mutants (34), P*_abcR1_*-derived fluorescence was 3- to 6-fold higher than that derived from P*_abcR1–38_*. In contrast, transcription from P*_abcR2_* specifically decreased in the *rpoH1* mutant to the basal levels rendered by P*_abcR2–38_*. The strongly reduced activity of P*_abcR2–38_*, relative to that of P*_abcR2_*, in the wild-type and mutant backgrounds is likely due to a deletion of transcriptional enhancers located upstream of many RpoH boxes, which remain uncharacterized in S. meliloti (34).

10.1128/mbio.03576-21.7 TABLE S1 Bacterial strains and plasmids. Download Table S1, DOCX file, 0.02 MB. Copyright © 2022 García-Tomsig et al. 2022 García-Tomsig et al. https://creativecommons.org/licenses/by/4.0/ This content is distributed under the terms of the Creative Commons Attribution 4.0 International license.

In an independent series of experiments, we used the same set of S. meliloti strains to examine AbcR1 and AbcR2 accumulation in different media and growth conditions by Northern blotting probing of total RNA with an oligonucleotide that cross-reacts with both transcripts (Fig. 1B and C). Hybridization of RNA from Sm2011 confirmed high levels of AbcR1 during exponential growth and an increased abundance of AbcR2 upon an osmotic upshift in both complete TY medium and defined minimal medium (MM) (Fig. 1B). In contrast, whereas AbcR2 retained its stress-induced expression in SmΔ*lsrB*, AbcR1 was undetectable in this mutant. In S. meliloti, RpoH1 and RpoH2 are coproduced at the onset of stationary-phase growth in MM and in response to a salt shock (34). Thus, we used these conditions to further assess RpoH-dependent expression of AbcR1/2 by Northern hybridization (Fig. 1C). Probing of RNA from strain Sm1021 confirmed AbcR2 expression during stationary phase and under salt stress. Unlike AbcR1, which accumulated in the absence of either σ^H^ factor, AbcR2 was not present in unstressed bacteria lacking RpoH1 and was downregulated, but reliably detected, in both *rpoH* mutants upon a salt shock. Therefore, it is likely that RpoH1 and RpoH2 contribute additively to AbcR2 transcription under this specific condition. This is not surprising given the similarities between the σ^H1^ and σ^H2^ motifs in S. meliloti promoters (34). To confirm this observation, we probed new RNA samples with an oligonucleotide specifically targeting AbcR2 (Fig. S1C). In this experiment, we also included the *rpoH1 rpoH2* double-insertion mutant, as well as RNA from bacteria subjected to a heat shock (40°C), which also promotes the activities of both σ^H^ factors. The lack of RpoH1 was enough to render AbcR2 undetectable under all conditions except salt stress, under which complete inhibition of AbcR2 expression required the double knockout. Together, these data revealed that the transcriptional regulation of AbcR1 and AbcR2 mostly depends on LsrB and RpoH1, respectively.

### MAPS-based characterization of the AbcR1/2 targetomes.

We used MAPS to identify the AbcR1/2 mRNA partners at a genome-wide scale. For this, we fused the MS2 RNA aptamer to the 5′ ends of AbcR1/2, which allows for the specific capture of the tagged transcripts along with their interacting mRNAs by an MS2-MBP (maltose-binding protein) fusion protein immobilized on an amylose resin. Tagging at the 5′ end was previously shown to preserve the stable expression and functional secondary structure of the AbcR sRNAs (35, 36). Given that AbcR1/2 and NfeR1 are predicted to share targets (14), wild-type AbcR1/2 (controls) and tagged AbcR1/2 were expressed from an isopropyl-β-d-thiogalactopyranoside (IPTG)-inducible promoter in the Δ*abcR1* Δ*abcR2* Δ*nfeR1* strain Sm2020 (an Sm2011 derivative). The sRNAs were reliably detected as transcripts of the expected sizes in total RNA extractions following 15, 30, and 60 min of IPTG addition (Fig. S2A). The chimeric transcripts also retained the ability to downregulate *prbA*, as shown using a *prbA*::*eGFP* translational fusion (Fig. S2B). These data validated the tagged sRNAs as baits for affinity chromatography.

10.1128/mbio.03576-21.3 FIG S2 MAPS setup. (A) Northern blot detection of MS2-AbcR1/2. RNA was obtained from S. meliloti Sm2020 transformed with pSKiAbcR1/2 or pSKiMS2AbcR1/2 0, 15, 30, and 60 min after the addition of IPTG to cultures in MM and probed with PbAbcR1/2. 5S rRNA was probed as an RNA loading control. (B) Tagged AbcR1/2 retained the ability to regulate *prbA*. Bar graphs represent fluorescence of reporter strains coexpressing the *prbA*::*eGFP* translational fusion and AbcR1/2 or their tagged variants (plasmids pSKiAbcR1/2 or pSKiMS2AbcR1/2) upon IPTG induction (24 h). Plotted values correspond to means and SD of 18 fluorescence measurements normalized to the OD_600_ of the cultures (Fl/OD_600_). The asterisks over the bars indicate statistically significant differences at a *P *of <0.05. (C) Monitoring of affinity chromatography. Expression of wild-type and MS2-AbcR1/2 was induced for 15 min with IPTG in S. meliloti Sm2020 transformed with pSKiAbcR1/2 or pSKiMS2AbcR1/2. RNA from the input, flowthrough (FT), wash, and output chromatography fractions (as indicated at the top of the panels) was probed with PbAbcR1/2. 5S rRNA was probed as a control. IGV plots of AbcR1/2 recovered in the elution (output) fractions are shown below. (D) Known AbcR1/2 target mRNAs copurified with the tagged transcripts. IGV plots showing read coverage and recovery profiles of *prbA* and *SMa0495* mRNAs upon affinity chromatography with wild-type and tagged AbcR1/2 as baits. The transcription start site of each mRNA is indicated (+1). Download FIG S2, TIF file, 2.1 MB. Copyright © 2022 García-Tomsig et al. 2022 García-Tomsig et al. https://creativecommons.org/licenses/by/4.0/ This content is distributed under the terms of the Creative Commons Attribution 4.0 International license.

To maximize copurification of target mRNAs and targetome coverage, we induced the transcription of wild-type controls and MS2-sRNAs for a short time (15 min) under conditions that promote their endogenous expression, i.e., exponential growth in TY and MM for AbcR1, and stationary-phase growth (TY and MM) as well as heat and salt shocks (both in TY) for AbcR2. Lysates from pools of AbcR1 and AbcR2 cultures were then subjected to affinity chromatography. Hybridization of RNA from the input and output chromatography fractions showed that the baits were specifically retained by the MS2-MBP protein (Fig. S2C). Mapping of the sequencing reads from the eluted RNA to the S. meliloti reference genome (Sm1021) unequivocally demonstrated efficient recovery of the tagged sRNAs and copurification of the known *prbA* and *SMa0495* targets (22), as expected (Fig. S2C and D).

Upon normalization by coverage, we compared read counts derived from controls and MS2-AbcR1/2 mapping to those of four mRNA regions: (i) the full-length mRNA, including the coding sequence and a virtual 5′ untranscribed region (5′-UTR) of 50 nt, (ii) a stretch of the 5′ region extending from nucleotide positions −50 to +100 relative to the translation start codon, (iii) the coding sequence alone, and (iv) the 3′ region encompassing 50 nt upstream and 30 nt downstream of the stop codon as a virtual 3′-UTR. We imposed a minimum of 50 (MS2-AbcR1) or 25 (MS2-AbcR2) mapped sequencing reads for an mRNA region to be considered in the comparisons. An mRNA was scored as a putative AbcR1/2 target if counts from tagged sRNA libraries exceeded a 3-fold difference (log_2_ fold change [log_2_FC] > 1.5) from counts for the controls in at least one of the computed regions. IntaRNA-predicted antisense interactions using the tagged sRNAs as queries were then used to filter out those mRNAs likely captured by unspecific binding to the MS2 aptamer. All three previously identified AbcR1/2 target mRNAs (*livK*, *prbA*, and *SMa0495*) passed the selection thresholds. All in all, MAPS identified 225 and 356 interacting mRNAs for AbcR1 and AbcR2, respectively, representing roughly 4% to 6% of S. meliloti protein-coding genes (Data Set S1).

10.1128/mbio.03576-21.9 DATA SET S1 MAPS data analysis. Download Data Set S1, XLSX file, 2.7 MB. Copyright © 2022 García-Tomsig et al. 2022 García-Tomsig et al. https://creativecommons.org/licenses/by/4.0/ This content is distributed under the terms of the Creative Commons Attribution 4.0 International license.

### AbcR1/2 broadly regulate S. meliloti metabolism.

**FIG 2:**
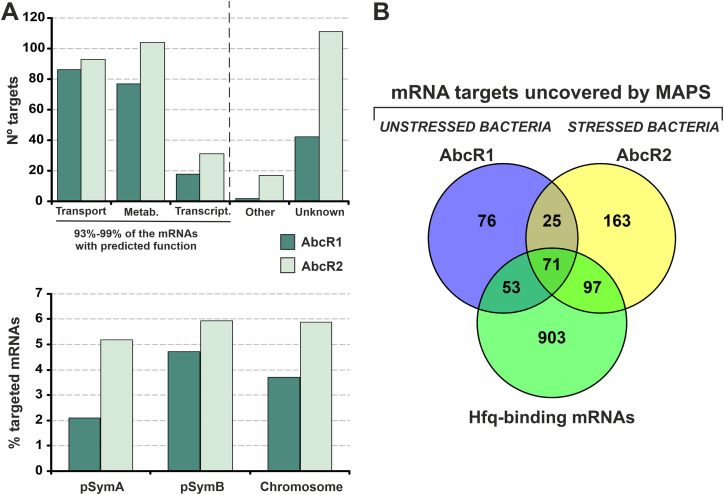
Overview of the AbcR1/2 mRNA interactomes determined by MAPS. (A) Functional categories of the captured mRNAs (top) and their distribution relative to the total number of protein-coding genes in each of the three S. meliloti replicons: the pSymA and pSymB megaplasmids and the chromosome (bottom). Metab., metabolism mRNAs; Transcript., transcription mRNAs. (B) Venn diagram comparing the AbcR1/2 targetomes and the set of known Hfq-binding mRNAs.

According to the Sm1021 genomic sequence annotation (37), 70% to 80% of the AbcR1/2 target mRNA candidates encode proteins with predicted functions (Fig. 2A). Of those, 72% (AbcR1) and 55% (AbcR2) are most probably involved in the transport or metabolism of widely diverse substrates. An additional 8% to 9% of both targetomes encode transcription factors, many of which are linked to metabolic operons. Collectively, the sets of metabolism-associated mRNAs represent 93% to 99% of the AbcR1 and AbcR2 targets with functional homology in database entries. The relative distribution of both targetomes along the three S. meliloti replicons indicates that the impact of AbcR1/2-mediated posttranscriptional regulation is slightly biased toward the pSymB megaplasmid (Fig. 2A), which is enriched in metabolic genes. Furthermore, pangenome analysis of 23 complete S. meliloti genomes identified 153 (68% of the total) and 215 (60.4%) AbcR1 and AbcR2 targets, respectively, as belonging to the core genome (Data Set S1). Although the different experimental setups (i.e., growth conditions) preclude a rigorous comparison between the AbcR1 and AbcR2 targetomes, MAPS uncovered 96 common targets for both sRNAs (Fig. 2B). Interestingly, 221 (20%) of the 1,127 mRNAs previously identified as Hfq ligands were also scored as AbcR1/2 targets (22), which indicates a prominent role of AbcR1/2 in the regulation of the extensive S. meliloti Hfq posttranscriptional network. The partial overlap between the AbcR1/2 targetomes and the Hfq partners suggests that most of these mRNAs are true sRNA targets rather than transcripts recovered unspecifically solely by binding to Hfq. This analysis thus anticipates a pervasive AbcR1/2 regulation of S. meliloti’s adaptive metabolism.

### AbcR1/2 use two distinct aSD motifs for regulation by base pairing.

**FIG 3:**
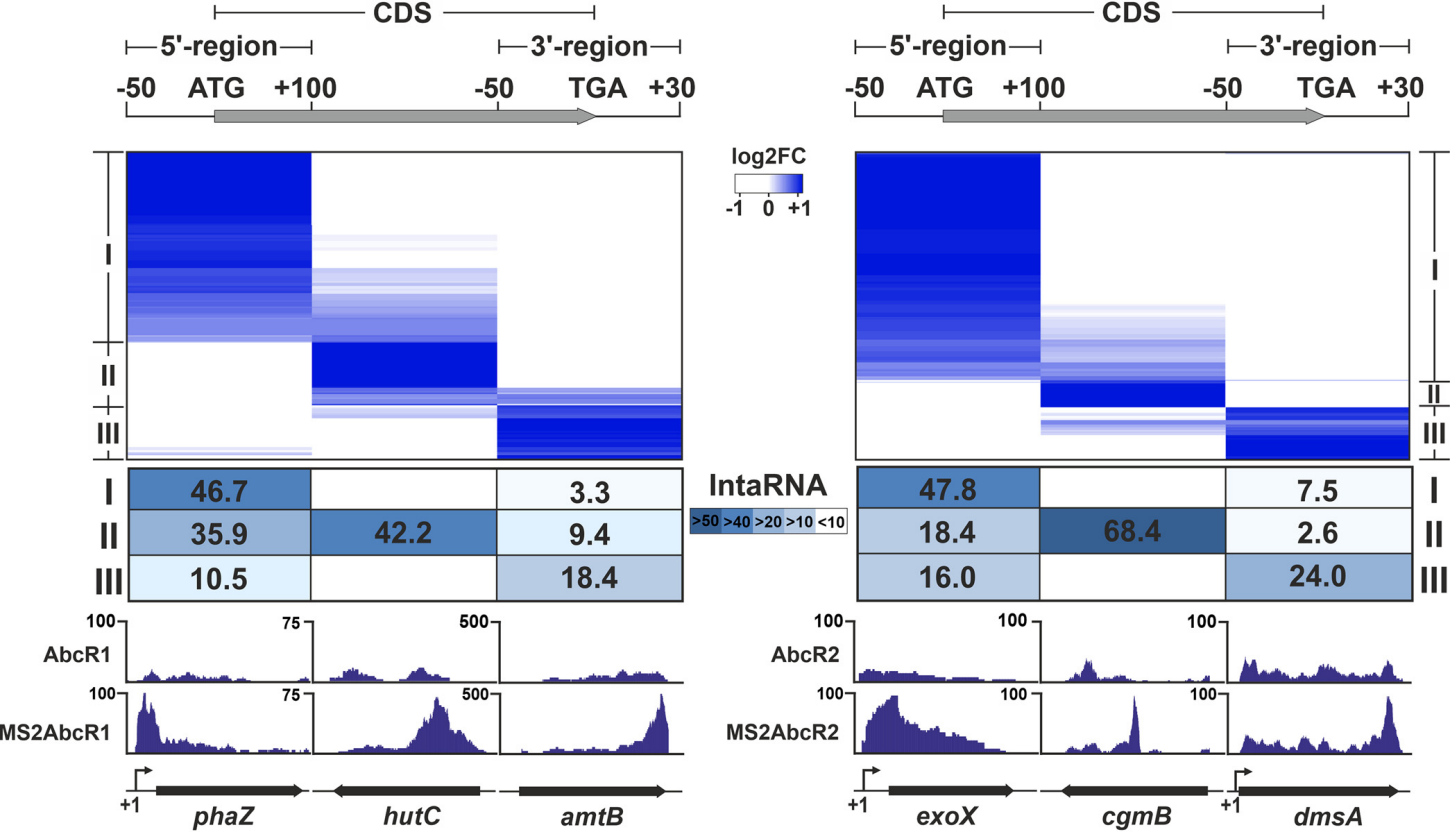
Enrichment-based clustering of target mRNAs upon affinity chromatography with MS2-AbcR1/2. Data for AbcR1 and AbcR2 are shown on the left and the right, respectively. Heatmaps identify three groups of mRNAs enriched at the 5′ region (cluster I), coding sequence (CDS; cluster II), and 3′ region (cluster III) with respect to the control experiments with the wild-type sRNAs (log_2_FC scale); these three regions are shown schematically at the top of the figure. The S. meliloti genome was interrogated with IntaRNA for thermodynamically favored (*E* < −8) antisense interactions (minimum, 7-nt seed) of AbcR1/2 in each of the mRNA regions. Numbers in the table indicate the percentage of mRNAs in each cluster that have a predicted antisense interaction within each region and is presented as a heatmap (legend between tables). Interactions predicted in the CDS may overlap those at the 5′ or 3′ mRNA region, and therefore, numbers in columns and rows may add up to more than 100%. IGV plots at the bottom show read coverages of target mRNAs representatives of each cluster.

Enrichment-based clustering of the mRNAs copurified with tagged AbcR1/2 unveiled three groups of targets, which were characterized by sequencing coverage biases toward either the 5′ region (cluster I), the coding sequence (cluster II), or the 3′ region (cluster III) (Fig. 3). Cluster I was the dominant in both targetomes. IntaRNA predictions revealed a correlation between the enrichment of a specific mRNA region and the location of the expected antisense AbcR1/2 interaction sites (Fig. 3). The target mRNAs *livK*, *prbA*, and *SMa0495* are representatives of the dominant cluster, cluster I. They were previously validated by means of translational fusions of their 5′ regions to *eGFP* as reporters of AbcR1/2-dependent regulation (15, 22). Here, we used a similar genetic reporter assay to validate a new set of three target mRNA candidates within cluster I that encode transport proteins: *SMc02417*, *SMc03121*, and *SMa0392* (Fig. S3A). IPTG-induced (over)expression of AbcR1/2 reduced fluorescence from the three reporters significantly, indicating downregulation of translation and equivalent regulatory abilities of the two sRNAs.

10.1128/mbio.03576-21.4 FIG S3 Experimental validation of newly identified target mRNAs and genetic dissection of the regulatory interactions. (A) Copurification of target mRNAs with AbcR1/2 and fluorescence reporter assays. IGV plots of *SMc02417*, *SMc03121*, and *SMa0392* coverage upon affinity chromatography with wild-type and MS2-AbcR1/2. Annotated transcription start sites of mRNAs are indicated (+1). Bar graphs below represent the fluorescence of strains coexpressing the target reporters indicated at the bottom and AbcR1/2 in noninduced (100% fluorescence) and IPTG-induced (24 h) cells. Asterisks indicate statistically significant differences at a *P *of <0.05. (B) Regulation of *SMa0392*, *SMc02417*, and *prbA* by aSD2. Diagrams depict the predicted base-pairing interactions between AbcR1/2 and each mRNA, with an indication of the hybridization energy (*E*). Numbers indicate nucleotide positions relative to the AUG start codon of the target mRNA (underlined) or the 5′ end of the sRNA. Nucleotide substitutions in AbcR1/2 (a/b variants) and target mRNAs (1/2 variants) are indicated. Nucleotides in red denote an alternative interaction between AbcR1/2 and *prbA*. The basal fluorescence of each reporter in noninduced bacteria (–IPTG) normalized by the OD_600_ of the cultures (Fl/OD_600_) is presented in the blue bar graphs. Red/green bars to the right report the rates that this basal fluorescence increased or decreased (target repression) upon IPTG induction of sRNA expression (24 h) in strains coexpressing the target reporters with wild-type AbcR1/2 or their mutant variants, as indicated at the bottom. Plotted values correspond to means and SD of 18 fluorescence measurements, i.e., three replicates in six double transconjugants for each reporter strain. Letters above/below the bars indicate statistical groups among values from assays with each target mRNA (groups of compared values are demarcated by the red lines; ANOVA test, *P < *0.05). Arrows and the double arrowhead over the bars stand for wild-type and restored non-wild-type regulation, respectively. Red bars indicate no regulation. Fluorescence patterns of the *prbA* reporters are compatible with target regulation by interaction of aSD2 at the two predicted sites (red and black arrows). Download FIG S3, TIF file, 2.3 MB. Copyright © 2022 García-Tomsig et al. 2022 García-Tomsig et al. https://creativecommons.org/licenses/by/4.0/ This content is distributed under the terms of the Creative Commons Attribution 4.0 International license.

**FIG 4:**
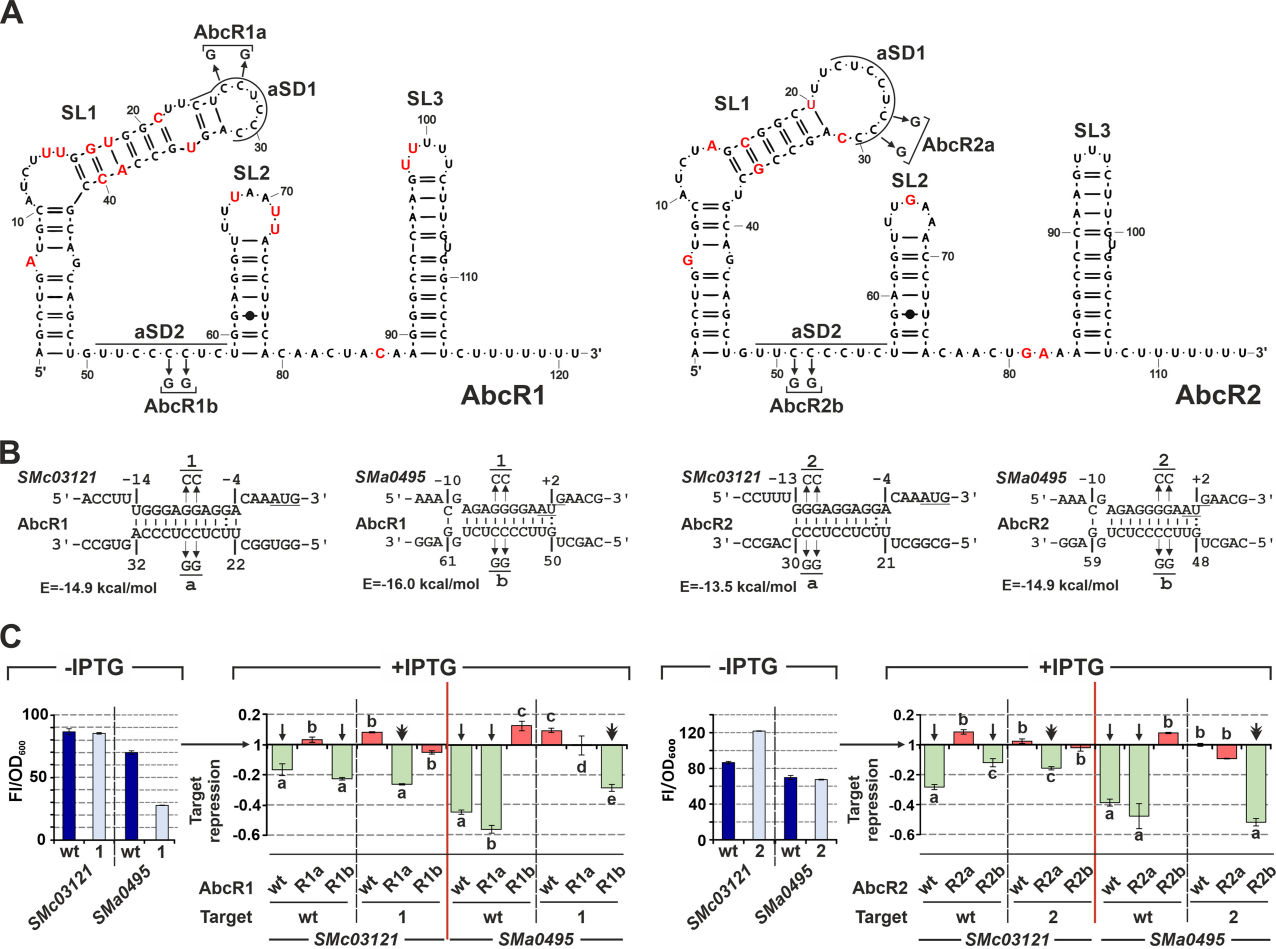
Genetic dissection of AbcR1/2 mRNA base-pairing interactions. (A) Predicted AbcR1 (left) and AbcR2 (right) secondary structures. Numbers indicate nucleotide positions relative to the 5′ end of each transcript. Stem-loops (SL) and the aSD-targeting motifs are indicated. Nucleotides differing between the sRNAs are indicated in red, while the substitutions in aSD1/2 are indicated by arrows. (B) Predicted base-pairing interactions between AbcR1/2 and the *SMc03121* or *SMa0495* mRNA. Numbers denote nucleotide positions relative to the AUG start codon (underlined) of the target mRNA or the 5′ end of the sRNA. The hybridization energy (*E*) and nucleotide substitutions in AbcR1/2 (a/b variants) and target mRNAs (1/2 variants) are indicated. (C) Fluorescence reporter assays. Fluorescence of each reporter (wild-type and variant 1 or 2) in noninduced bacteria (without IPTG [–IPTG]) normalized by the OD_600_ of the cultures (Fl/OD_600_) is presented in the blue bar graphs. The red/green bars to the right report the rates at which this basal fluorescence increased or decreased (target repression) upon IPTG induction of sRNA expression (24 h) in strains coexpressing the target reporters with wild-type (wt) AbcR1/2 or their mutant variants, as indicated at the bottom. Plotted values correspond to means and SD of 18 fluorescence measurements, i.e., from three replicates of six double transconjugants for each reporter strain. Letters above/below bars indicate statistical groups among values from assays with each target mRNA (groups of compared values are demarcated by the red lines; analysis of variance [ANOVA] test, *P < *0.05). Arrows and the double arrowhead over the bars indicate the wild-type and restored non-wild-type regulation, respectively. Red bars represent no regulation.

The overlapping targeting potential of AbcR1/2 likely relies on identical pairs of 8-nt aSD motifs located at the loop of the 5′ hairpin (CUCCUCCC; aSD1) and between SL1 and SL2 stem loops (UUCCCCUC; aSD2) in both molecules (Fig. 4A). To pinpoint the contribution of aSD1 and aSD2 to regulation, we first assessed the activities of two AbcR1/2 variants on the *SMc03121* and *SMa0495* targets using the translational reporter fusion assay. Specifically, we introduced 2-nucleotide substitutions within either aSD1 (AbcR1a/AbcR2a) or aSD2 (AbcR1b/AbcR2b) that preserve the putative secondary structures of both transcripts while disrupting the predicted base pairing at the translation initiation region of the target mRNAs (Fig. 4A and B). Interaction with *SMc03121* probably occurs via aSD1, whereas *SMa0495* is likely targeted by aSD2 (Fig. 4B). Induced (over)expression of wild-type AbcR1/2 resulted in a decrease of reporter-derived fluorescence in both cases. Consistently with the predicted interactions, AbcR1/2a variants (aSD1 mutants) retained wild-type activity on *SMa0495* but lost the ability to repress *SMc03121*, whereas the regulatory effects were the opposite with mutants in aSD2 (Fig. 4C). *SMc03121* is thus a common target of NfeR1 and AbcR1/2, which is regulated by interactions of aSD seeds at the RBS. Nucleotide changes in *SMc03121* and *SMa0495* leaders compensating for mutations in AbcR1/2 that abrogated target regulation did not inhibit the translation of the *eGFP* reporter (Fig. 4C, blue bars). However, *SMa0495-1* (complementary to AbcR1b) decreased the basal fluorescence of the fusion to ∼40% of the wild type, most likely by interfering with the SD sequence. Remarkably, all these nucleotide substitutions fully abrogated the activities of wild-type AbcR1/2, while restoring regulation by the complementary variants (Fig. 4C). A similar genetic dissection revealed *SMa0392*, *SMc02417*, and *prbA* regulation via aSD2 (Fig. S3B). *SMa0392* leaders with nucleotide changes that restore pairing with AbcR1b/2b reduced basal-level expression of the reporters by 75 to 50% and were not regulated by the wild-type sRNAs. These mutations supported the regulation by AbcR1b/2b, as expected. Consistently with disruption of the RBS, point mutations at the predicted AbcR1/2 binding sites in *SMc02417* inhibited translation (i.e., the basal activities of the reporters were scarcely 4% of the wild-type activity), thus precluding further unambiguous confirmation of aSD2 interaction at these sites. Finally, fluorescence patterns of wild-type and mutant *prbA* reporters suggest regulation by AbcR1/2 via aSD2 pairing at two contiguous sites in the mRNA leader. Wild-type sRNAs were fully active on *prbA* mutants, disrupting pairing with aSD2 in only one of these sites. However, these mutations were sufficient to restore regulation by AbcR1b/2b. This redundant targeting is reminiscent of *lrp* (leucine-responsive regulatory protein) regulation by GcvB in Escherichia coli (38). Collectively, these findings indicate that aSD1 and aSD2 are independent base pairing-targeting motifs that may be designed to regulate noncognate AbcR1/2 target mRNAs.

### Metabolic-model-aided analysis of the AbcR1/2 targetomes.

**FIG 5:**
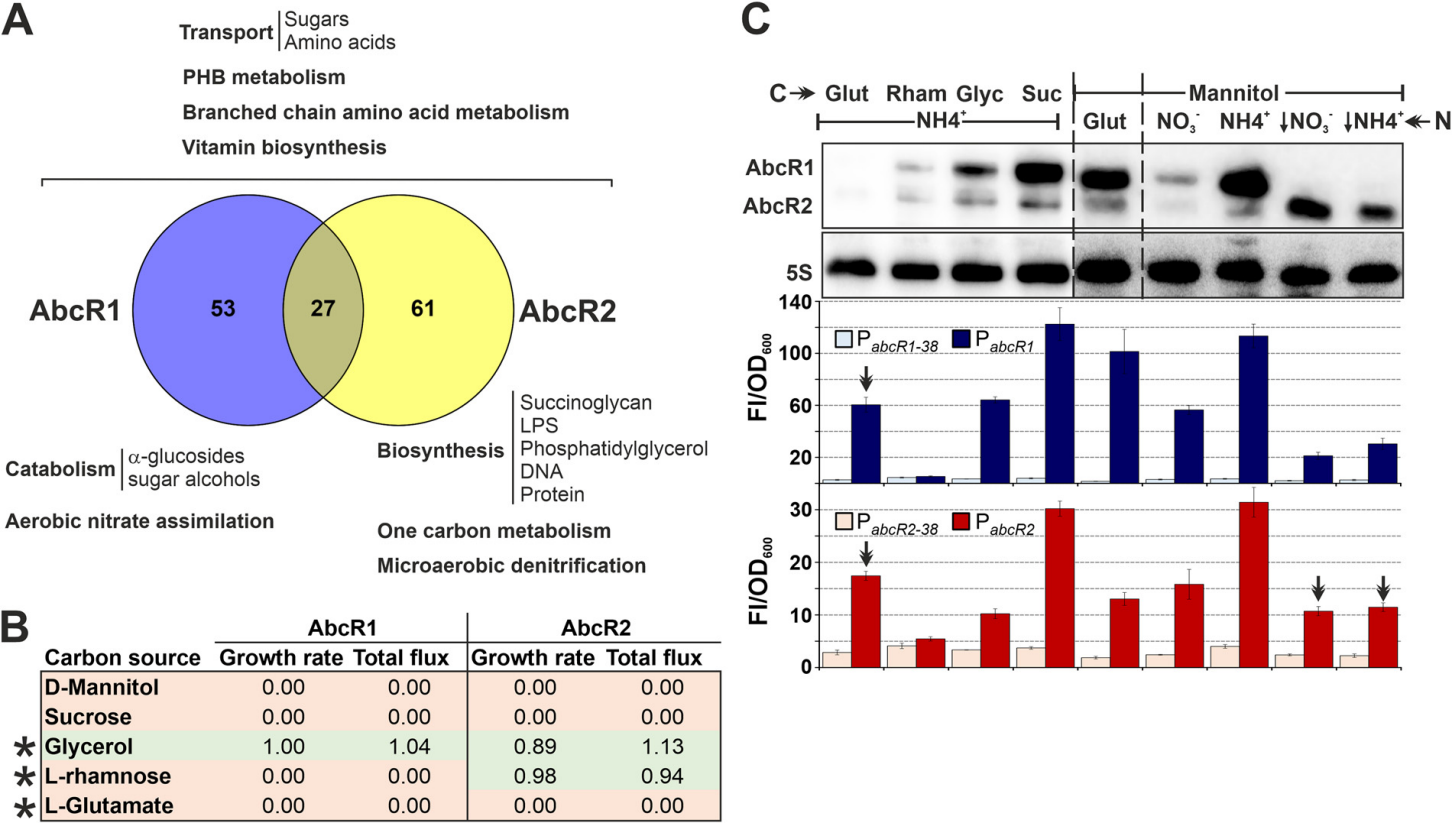
Metabolic model-assisted analysis of the AbcR1/2 targetomes. (A) Major transport/metabolic reactions likely regulated by AbcR1/2. The Venn diagram compares the numbers of AbcR1/2 target mRNAs represented in the model. (B) Predicted impact of target gene deletions in growth/metabolic flux in simulated defined media. Carbon sources in the media are indicated. Cells shaded red stand for the maximum impact, and those in green indicate little to no impact. Asterisks indicate matches between predictions and experimental results. (C) Changes in AbcR1/2 expression driven by shifts in carbon and nitrogen sources. Northern blot probing of total RNA from the S. meliloti Sm2B3001 strain upon growth to the onset of stationary phase in defined media with the carbon and nitrogen substrates indicated along the top. AbcR1/2 levels in mannitol-glutamate MM are considered the reference. Arrows indicate nitrogen stress imposed with a 0.5 mM concentration of either nitrate or ammonia. The 5S rRNA was probed as an RNA loading control. Shown is the hybridization corresponding to one of two biological replicates with identical results. Bar graphs below represent fluorescence values from promoter-*eGFP* fusions under each growth condition determined as described in Fig. 1A. Double arrowheads indicate conditions that presumably promote AbcR1/2 posttranscriptional regulation.

To further delineate AbcR1/2 function, we linked their regulons to the S. meliloti genome-scale metabolic model iGD1348, which combines core and accessory transport/metabolic reactions specified by 1,348 protein coding genes (39). Eighty (35%) and 88 (25%) AbcR1 and AbcR2 targets, respectively, are represented in this model, with 27 belonging to both targetomes (Fig. 5A; Data Set S1). Traits likely regulated by both sRNAs are the uptake of diverse sugars and amino acids, polyhydroxybutyrate (PHB) and branched-chain amino acids (BCAAs) metabolism, and vitamin biosynthesis. AbcR1 seems to specifically regulate the catabolism of α-glucosides and sugar alcohols and the aerobic assimilation of nitrate in rich media. One-carbon metabolism, microaerobic denitrification, and the biosynthesis of succinoglycan (exopolysaccharide [EPS]), lipopolysaccharide (LPS), or phosphatidylglycerol are major pathways influenced by AbcR2 under abiotic stress.

We next used flux balance analysis (FBA) to predict the impact of AbcR1/2 target deletion on bacterial growth and parsimonious FBA (pFBA) to predict the requirement of a particular gene for optimal flux patterns (i.e., the total metabolic flux rate). The consequences of gene deletion were examined in simulated defined media differing in carbon substrates while keeping ammonia as the nitrogen source. A change of at least 10% in growth rate or total flux was considered significant (Data Set S1). Overall, these simulations predict that the combined AbcR1/2 regulon influences S. meliloti transport/metabolism during growth with 64 of the 83 (77%) tested carbon substrates.

Since AbcR1/2 promote posttranscriptional silencing, we expected downregulation of the sRNAs if one or more of their target mRNAs were predicted to be essential for optimal growth with a defined carbon substrate, e.g., mannitol, sucrose, glycerol, rhamnose, or glutamate (Fig. 5B). Probing of RNA from bacteria cultured in these media confirmed the predicted downregulation of AbcR1/2 with rhamnose and glutamate, but not with mannitol or sucrose; predictions support the observed AbcR1/2 expression with glycerol (Fig. 5C). The apparent discrepancies between *in silico* and experimental data are likely due to the model assuming a complete loss rather than a fine-tuning of target gene expression and/or model incompleteness (i.e., transcription factors are not included, and genes specifying putative redundant transport/metabolic reactions might be missing). Although the model was not used to interrogate nitrogen metabolism, we found downregulation of both sRNAs under nitrate surplus. Nitrogen stress imposed with either ammonia or nitrate prevented AbcR1 expression while promoting AbcR2 accumulation (Fig. 5C). Fluorescence of promoter-reporter fusions revealed an overall correlation between the strength of transcription and AbcR1/2 steady-state levels under each growth condition (Fig. 5C). Exceptions were growth in glutamate/ammonia and nitrogen stress, thus hinting at posttranscriptional AbcR1/2 regulation under these conditions.

**FIG 6:**
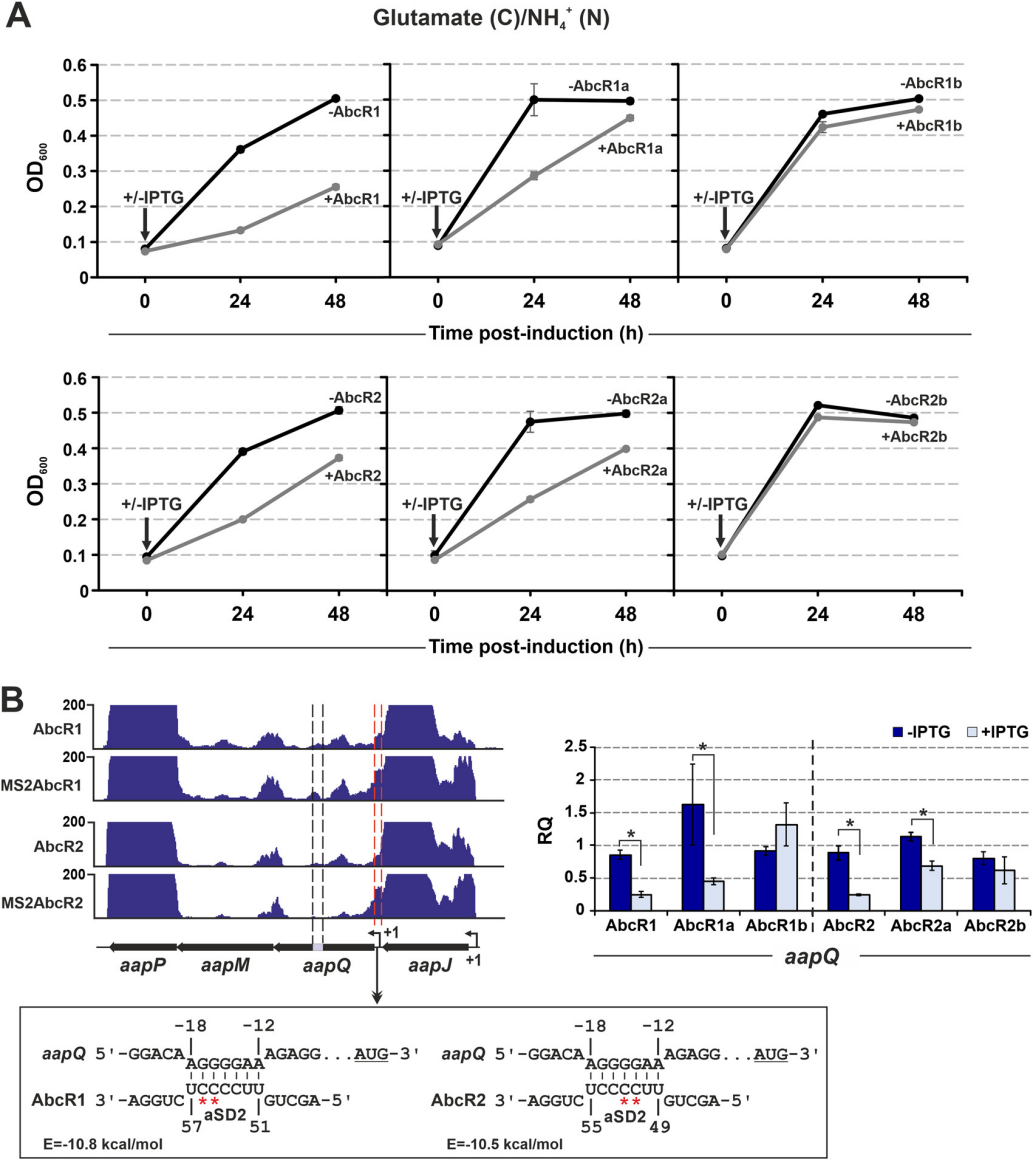
AbcR1/2 silence the mRNA coding for the l-amino acid permease AapQ. (A) AbcR1/2 overexpression growth phenotype. Growth kinetics of the S. meliloti Sm2020 strain transformed with plasmids (over)expressing wild-type AbcR1/2 or their aSD1/2 mutants from an IPTG-inducible promoter. Bacteria were grown in defined glutamate-ammonia media to an OD_600_ of 0.1, at which point sRNA expression was either induced (IPTG addition) or left uninduced (no IPTG). The OD_600_ was recorded for the induced and uninduced cultures 24 and 48 h following IPTG addition. Plotted values are means and SD from three independent experiments. (B) RT-qPCR analysis of *aapQ* regulation. (Left) IGV images of the affinity purification recovery profiles of *aapJQMP*. *aapQ* regions enriched with respect to control experiments and amplified using RT-qPCR are demarcated by red and black dashed lines, respectively. The diagrams below depict the predicted base-pairing interactions of AbcR1/2 at the translation initiation region of *aapQ*, with indication of the hybridization energy (*E*). Numbers indicate nucleotide positions relative to the *aapQ* AUG start codon (underlined) or the AbcR1/2 5′ ends. The red asterisks denote the nucleotides replaced in the aSD2 AbcR1/2 motif (AbcR1/2b variants). (Right) RT-qPCR analysis of *aapQ* abundance 30 min after inducing the expression of wild-type AbcR1/2 or their mutant variants by IPTG addition to glutamate-ammonia cultures. Relative quantification (RQ) values were normalized to those for *SMc01852* as a constitutive control. Values plotted in the bar graphs are means and standard errors (SE) from three replicates of three biological replicates. Asterisks above the bars indicate statistically significant differences at a *P *of <0.05.

Modeling analysis predicts that AbcR1/2 expression may limit S. meliloti growth in glutamate/ammonia medium by silencing the l-amino acid ABC transporter AapJQMP. Growth kinetics in this medium confirmed that the growth rate of strain Sm2020 was reduced upon IPTG-induced expression of AbcR1/2 or their AbcR1/2a variants, but not with AbcR1/2b (Fig. 6A). Scanning of *aapJQMP* with IntaRNA for base pairing to AbcR1/2 unveiled a thermodynamically favored interaction (hybridization energy [*E*] < 8 kcal/mol) with the aSD2 seed 12 nt upstream of the start codon of *aapQ*, which encodes the permease of the system (Fig. 6B). This was consistent with the MAPS profiles, which suggested that this interaction might promote *aapQ* decay. Reverse transcriptase quantitative PCR (RT-qPCR) of RNA extracts from a similar growth experiment confirmed AbcR1/2-dependent *aapQ* depletion through aSD2 (Fig. 6B). All together, these data support the finding that AbcR1/2 selectively silences S. meliloti transport/metabolic mRNAs in response to shifts in both carbon and nitrogen substrates.

### AbcR1 is required for wild-type colonization of alfalfa roots.

**FIG 7:**
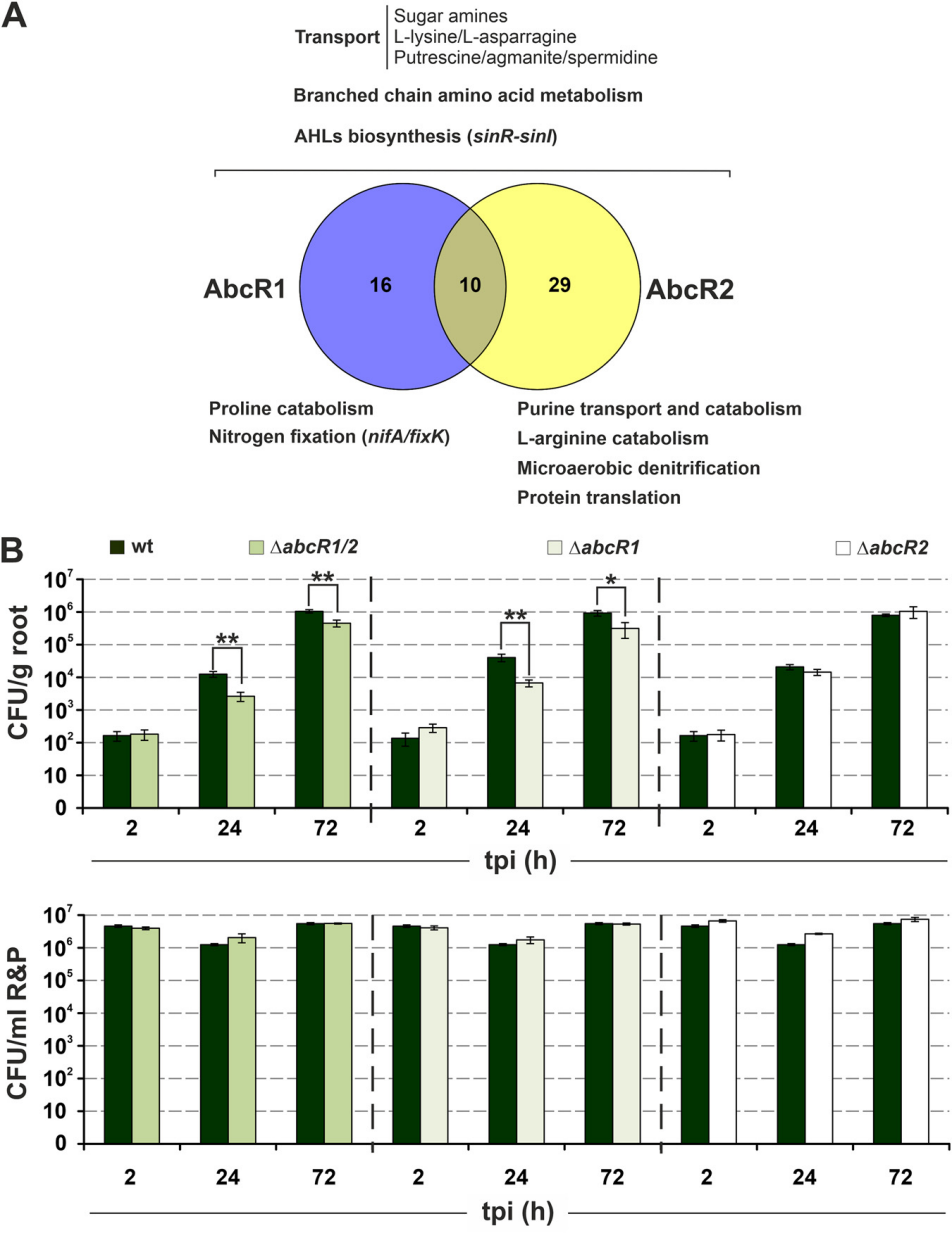
AbcR1 contributes to alfalfa root colonization. (A) Overlap between the AbcR1/2 targetomes and S. meliloti transcriptomic signatures under rhizosphere-like conditions. Major transport/metabolic reactions likely regulated by AbcR1/2 and relevant to rhizosphere colonization are indicated. (B) Root colonization assay. Alfalfa plants grown hydroponically were inoculated with either the wild-type Sm2B3001 strain or its single or double *abcR1/2* deletion mutant as indicated along the top. Bar graphs represent the number of bacteria released from roots (CFU per gram of root [upper panel]) or remaining in the R&P rooting solution (CFU per milliliter [lower panel]) at different times postinoculation (tpi). Values are means and SD of counts for a total of 45 roots from plants inoculated with each strain (three sets of 15 plants per strain treated independently). * and ** above the bars indicate statistically significant differences at *P *values of <0.05 and <0.005, respectively.

Expression profiles suggest a prevalent AbcR1/2 activity in free-living rhizobia colonizing bulk soil or the legume rhizosphere but not in endosymbiotic bacteroids (15). Database searches identified several clusters of AbcR1/2-interacting mRNAs that are differentially expressed under rhizosphere-related conditions (i.e., exposure to alfalfa root exudates or to the nodulation gene inducer luteolin) (Fig. S4; Data Set S1). These mRNAs specify well-recognized S. meliloti metabolic traits for efficient colonization of the alfalfa rhizosphere, for example, transport/metabolism of diverse amino acids and other complex nitrogen sources and biosynthesis of the quorum-sensing autoinducers *N*-acyl-homoserine lactones (AHLs) (Fig. 7A). A CopraRNA-based survey of a set of phylogenetically related genomes predicts that the regulation of orthologs of the AbcR1 target mRNAs belonging to the S. meliloti core genome is conserved across alphaproteobacteria interacting with eukaryotic hosts (Fig. S5). However, the occurrence of rhizosphere-related mRNA orthologs and their regulation by AbcR1 is limited to legume symbionts and even more constrained to close S. meliloti relatives in the case of target genes belonging to the S. meliloti accessory genome. We obtained a similar picture when AbcR2 sequences were used as queries (not shown). This conservation pattern suggests that the AbcR1/2 regulon has evolved to help alphaproteobacteria colonize the host-specific environment. These findings prompted us to investigate the impact of AbcR1/2 on the ability of S. meliloti to proliferate on the root rhizoplane during preinfection stages of symbiosis with alfalfa.

10.1128/mbio.03576-21.5 FIG S4 Occurrence of the AbcR1/2 target mRNAs in rhizosphere-related S. meliloti transcriptomic signatures. Heatmaps of expression data from S. meliloti Sm1021 cultures treated with luteolin and root exudates of three alfalfa varieties (Verbena, Camporegio, and Lodi). Clustering is performed on Euclidean distances using the average distance method. Download FIG S4, TIF file, 1.0 MB. Copyright © 2022 García-Tomsig et al. 2022 García-Tomsig et al. https://creativecommons.org/licenses/by/4.0/ This content is distributed under the terms of the Creative Commons Attribution 4.0 International license.

10.1128/mbio.03576-21.6 FIG S5 Conservation of AbcR1 regulation in alphaproteobacteria. AbcR1 homologs in S. meliloti Sm1021, *S. medicae* WSM419, *S. fredii* HH103, R. leguminosarum bv. viciae 3841, R. leguminosarum bv. trifolii WSM2304, *R. etli* CIAT652, A. tumefaciens C58, and B. abortus 2308 were used as queries to interrogate the corresponding genome with CopraRNA for conserved base pairing at the 5′ regions of the annotated mRNAs (positions −100/+300 relative to the annotated start codons). The heatmap represents conservation of the indicated set of core/accessory/rhizosphere S. meliloti mRNAs identified by MAPS (rows) and their base-pairing interactions with AbcR1. Columns are ordered according to an unweighted pair group method using average linkages (UPGMA) tree based on the homology of AbcR1 sequences. Cells are colored based on the respective IntaRNA *P* value, as indicated, i.e., the probability of the predicted base pairing in each individual genome. White cells indicate that the target mRNA is absent in the given bacterium according to the DomClust clustering of CopraRNA. Download FIG S5, TIF file, 0.8 MB. Copyright © 2022 García-Tomsig et al. 2022 García-Tomsig et al. https://creativecommons.org/licenses/by/4.0/ This content is distributed under the terms of the Creative Commons Attribution 4.0 International license.

We inoculated sets of alfalfa plants grown hydroponically with equivalent cell densities (10^6^ cells/mL rooting solution) of the wild-type S. meliloti Sm2B3001 strain (Sm2011 in which the *expR* gene was restored) or single or double AbcR1/2 deletion mutants. Bacterial populations either attaching to roots (cells/gram of root) or remaining in the rooting solution were then monitored by plate counting at 2, 24, and 72 h after plant inoculation (Fig. 7B). Counts remained invariable and equivalent among strains in the rooting solution throughout the experiment, indicating that the rooting medium does not support bacterial growth. Conversely, bacterial density on the root surface increased exponentially, indicative of active rhizoplane colonization supported by root exudates. Bacterial populations released from roots 24 and 72 h after inoculation were significantly lower in the AbcR1 and AbcR1/2 deletion mutants than in the wild-type strain, whereas a lack of AbcR2 did not influence colonization kinetics. FBA simulations with the metabolic S. meliloti model using a simulated rhizosphere environment similarly predicted that the AbcR1 targetome has a much greater influence on rhizosphere colonization than that of AbcR2 (Data Set S1). These data thus revealed a specific contribution of AbcR1 to alfalfa root colonization.

## DISCUSSION

**FIG 8:**
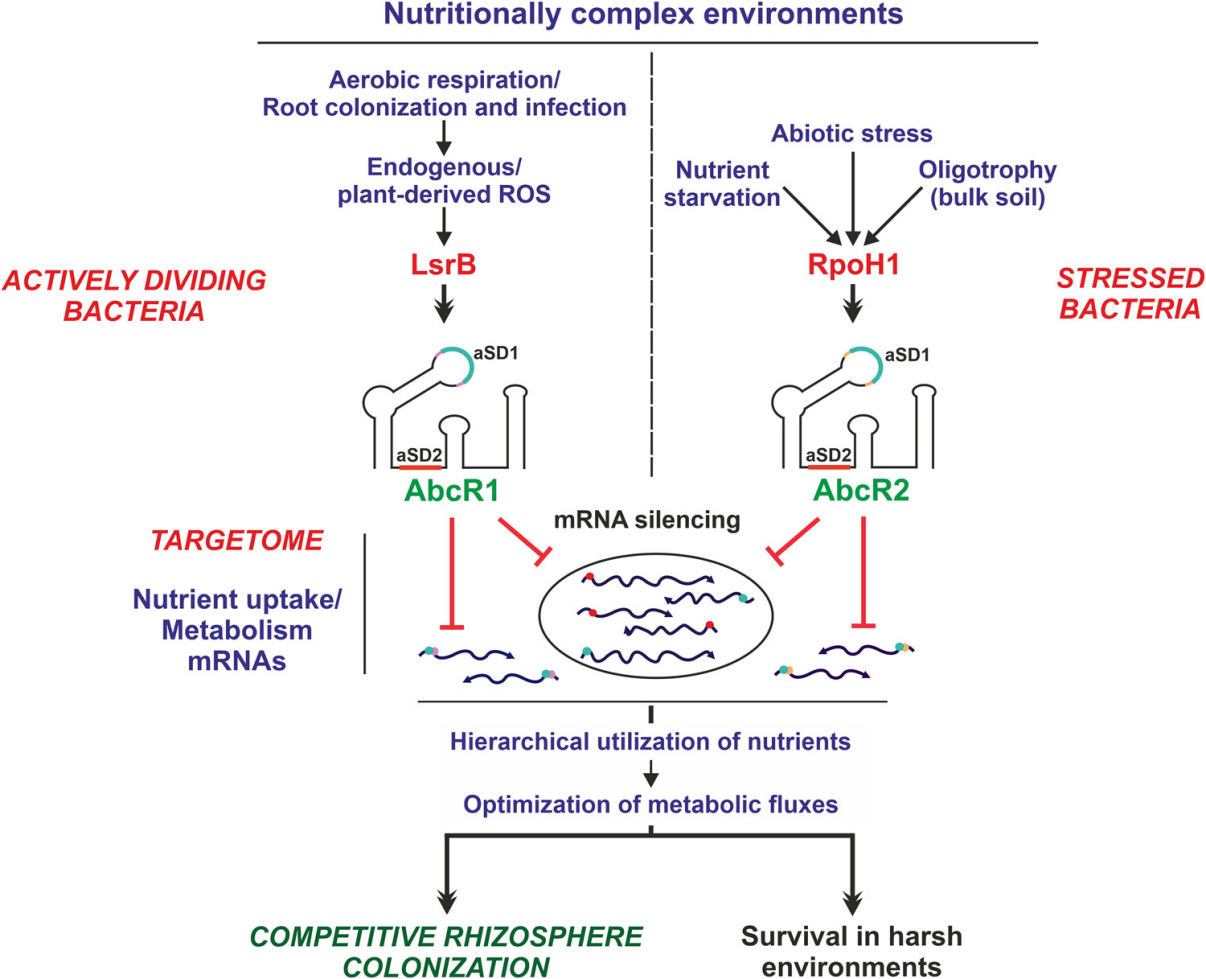
S. meliloti AbcR1/2 posttranscriptional regulatory network. Graphical summary of data. Details are in the text.

Base-pairing sRNAs have pivotal roles in fine-tuning the transcriptional output from regulatory networks that govern environmental adaptations in bacteria (40). However, regulation by RNA remains poorly investigated in most environmentally relevant microbes. Here, we show that the homologous S. meliloti *trans*-sRNAs AbcR1 and AbcR2 respond to metabolic and stress signals transduced via the LTTR LsrB and the alternative σ factor RpoH1, respectively, to silence large and overlapping arrays of mRNAs related to nutrient uptake and metabolism (Fig. 8). Remarkably, metabolic rewiring by AbcR1 optimizes S. meliloti’s ability to colonize the nutrient-rich root rhizoplane during early stages of symbiosis with its legume host. To the best of our knowledge, this is the first comprehensive genome-wide description of an RNA regulatory network controlling a major adaptive trait in nitrogen-fixing legume symbionts, a group of soil bacteria essential for planet sustainability.

### LsrB and RpoH1 trace independent input modules for the AbcR1/2 network.

sRNA abundance is regulated primarily at the level of transcription initiation (41). Supporting *in silico* predictions, genetic and biochemical approaches unequivocally identified LsrB with the σ factor RpoH1 as the major regulators of S. meliloti AbcR1 and AbcR2 transcription, respectively. These findings confirm that the regulation of AbcR1 is conserved in alphaproteobacteria (28–30). S. meliloti LsrB senses the concentration of reactive oxygen species (ROS) that may derive from either aerobic respiration (endogenous ROS), redox-cycling compounds secreted by neighboring soil organisms, or the oxidative bursts of plant defense responses during symbiosis (42, 43). Redox signal transduction by LsrB boosts the transcription of genes for the biosynthesis of LPS and ROS scavenging systems (e.g., glutathione), thereby preventing cell damage, root infection arrest, and premature nodule senescence (44, 45). Thus, plant-derived ROS is likely the biotic signal that drives AbcR1 transcription in undifferentiated rhizobia at early symbiotic stages (15, 23, 46). Our data further suggest that LsrB might also transduce shifts in carbon and nitrogen metabolism in free-living S. meliloti bacteria (Fig. 5C), possibly by sensing the differential accumulation of endogenous ROS. Unlike that of AbcR1, AbcR2 regulation had not previously been investigated in alphaproteobacteria. A microarray-based transcriptome profile of S. meliloti *rpoH* mutants revealed differential expression of a few annotated *trans*-sRNAs but overlooked the downregulation of AbcR2 (34). RpoH1 recognizes gene promoters that respond to diverse stressors, such as heat shock, salinity, nutrient starvation, or the plant intracellular milieu (34), which is consistent with the stress-induced transcription of AbcR2 in free-living bacteria (see Fig. S1 in the supplemental material).

In S. meliloti, a lack of either LsrB or RpoH1 results in severe growth and endosymbiotic phenotypes, hinting at their seemingly constitutive activity, which only partially explains the differential AbcR1/2 expression (e.g., AbcR2 is not detected in nodules) (34, 47, 48). Thus, posttranscriptional and/or posttranslational modifications of LsrB and RpoH1 might be further determinants of AbcR1/2 transcription rates in free-living and symbiotic rhizobia (49). In this regard, it is known that the strength of LsrB regulation depends on the oxidation of two cysteine residues that promote protein dimerization via disulfide bonds (42). The extent to which this posttranslational modification influences LsrB affinity for promoter binding and AbcR1 transcription must be investigated.

### MAPS-derived insights into the AbcR1/2 network.

The identification of regulatory motifs in *trans*-sRNAs and their mRNA targets remains challenging (50, 51). Computational tools typically predict large sets of target mRNA candidates for a *trans*-sRNA, but the limited complementarity between the partners often leads to exceedingly high false-positive prediction rates (52). This also applies to S. meliloti AbcR1/2, although previous genetic reporter assays confirmed as targets a few mRNA candidates predicted *in silico* (15, 22). Even though these assays are suitable to validate and dissect sRNA-mRNA interactions, they do not provide genome-wide insights into the regulation occurring endogenously under specific environmental conditions. Thus, we chose MAPS to tackle the comprehensive profiling of the S. meliloti AbcR1/2 mRNA interactomes under growth conditions that stimulate endogenous upregulation of each sRNA (53). Remarkably, roughly 6% of S. meliloti mRNAs were identified in the combined AbcR1/2 targetome, most of which encode nutrient uptake, catabolism, or biosynthesis functions (Data Set S1). AbcR1/2 thus resemble E. coli GcvB sRNA in regulating an exceptionally large number of metabolic genes (54, 55). AbcR1/2 are core components of the S. meliloti pangenome (24), but many of their putative mRNA targets are Hfq partners, belong to the accessory genome, and/or are encoded in the pSymB megaplasmid (Fig. 2). This suggests a major impact of these sRNAs in the effective integration of acquired adaptive metabolism into core regulatory networks (22, 56).

MAPS captures sRNA-mRNA base pairing, but it is not inherently designed to inform about the impact of these interactions on target mRNA stability. However, the markedly uneven distribution of sequencing reads over large sets of mRNAs copurified with tagged AbcR1/2 suggests accelerated decay of the targets upon base pairing, thereby providing interaction signatures for regulation (Fig. 3). In S. meliloti, the set of ribonucleases and degradosome-like assemblies containing Hfq are poorly characterized (13, 35, 57). Our MAPS setup might report on the turnover dynamics of the AbcR1/2 mRNA interactomes if sRNA baits are expressed in the relevant RNase knockout mutants.

Interaction signatures inferred from the recovery profiles of the AbcR1/2 target mRNAs and further genetic approaches suggest that AbcR1/2 act predominantly by a canonical Hfq-dependent mechanism relying on base pairing at the RBS leading to translation inhibition (21) (Fig. 3 and 4 and Fig. S3). Nonetheless, our data also envisage minor but plausible alternative modes of action independent of Hfq or involving interactions in the coding sequence of the target mRNAs (Fig. 3). The latter has already been shown for the A. tumefaciens AbcR1/2 homologs (31). Reporter assays confirmed AbcR1/2 regulation of a set of three newly identified targets (*SMc02417*, *SMc03121*, *SMa0392*) that all code for ABC transport proteins (Fig. S3). These experiments provided further evidence that the aSD seeds (aSD1/2) are major motifs involved in mRNA targeting. aSD1 and aSD2 differ slightly in their nucleotide sequences, and they were genetically shown to fulfil independent targeting roles (Fig. 4). A few of the nucleotides that flank aSD1 differ between AbcR1 and AbcR2, which might provide specificity for targeting. Indeed, we previously showed specific AbcR1-mediated silencing of *livK* most likely through aSD1 (15). Conversely, aSD2 is embedded within an ultraconserved nucleotide stretch and presumably supports the regulation of common target sets. Therefore, it seems likely that the functional specificity of AbcR1 and AbcR2 is conferred largely by their differential expression rather than by their targeting potential.

### A metabolic model delineated the adaptive functions of AbcR1/2.

In S. meliloti, many predicted transport or metabolic reactions have scarce experimental support. A metabolic-model-assisted analysis of the targetomes charted by MAPS suggested that AbcR1/2’s impact extends beyond primary carbon/nitrogen energy pathways to the regulation of biosynthesis of the major carbon storage polymer PHB or cell envelope components (e.g., EPS, LPS, phosphatidylglycerol) (Fig. 5). In S. meliloti, PHB biosynthesis is negatively regulated by the MmgR sRNA under carbon surplus conditions (19). MmgR is repressed by the global carbon flow regulator AniA (58, 59), which is a putative target of AbcR1/2 regulation. Thus, AniA may serve as a connection node of the MmgR and AbcR1/2 regulatory networks for the robust control of carbon homeostasis. mRNAs specifying cell wall synthesis were identified as AbcR2-specific targets. Therefore, AbcR2 might play a role in the regulation of cell envelope remodeling in response to different stresses (60–62). The model also linked AbcR1/2 targets to nitrate assimilation and denitrification pathways, which was further supported by profound changes in AbcR1/2 expression upon shifts in the quality and quantity of the nitrogen source (Fig. 5C). RNA regulation of nitrogen metabolism has been reported in free-living nitrogen fixers but not in S. meliloti (63–65).

This approach was useful in predicting AbcR1/2 targets required for growth in defined media formulated with specific carbon substrates. As a proof of principle, we demonstrated AbcR1/2-mediated silencing of the permease component of the l-amino acid transporter AapJQMP (Fig. 6). Together with the BraDEFGC transport system, AapJQMP rescues the symbiotic autotrophy for BCAAs of Rhizobium leguminosarum bv. viciae bacteroids within indeterminate pea nodules, which is not a feature of alfalfa nodules (66, 67). Regulation of *aapQ* by AbcR1/2 is predicted to be conserved in R. leguminosarum and may have a specific impact on pea nodule metabolism that merits further investigation.

### A target-centric perspective of AbcR1 contribution to root colonization.

Root exudates make the rhizosphere and rhizoplane nutrient-rich but strongly selective environments for the root microbiome (3, 68). The AbcR1 knockout phenotype thus suggests that RNA regulation of metabolism provides S. meliloti with a competitive advantage for host root colonization and saprophytic long-term survival in the rhizosphere. Both the reported specific contribution of AbcR1 to S. meliloti growth in complete media and this novel phenotype are consistent with AbcR1 levels far exceeding AbcR2 levels when both sRNAs are cotranscribed in rhizobia actively dividing under nutrient surplus or at preinfection symbiotic stages (15). Expression and targetome profiles of AbcR2 predict similar impacts of this sRNA in the colonization of bulk soil or the rhizosphere under harsh environmental conditions.

The AbcR1/2 interactomes are well represented in the transcriptomic signatures of rhizospheric S. meliloti bacteria (Fig. S4), which are enriched in transport/metabolic genes for the utilization of amino acids, sugar amines, and polyamines (69). Interestingly, mariner-based transposon insertion sequencing has recently uncovered that knockout of genes encoding uptake systems for quaternary amines, BCAAs, l-amino acids (e.g., *aapJQMP*), opines, and polyamines enhances R. leguminosarum bv. viciae fitness in pea rhizosphere (2). Similarly, downregulation of the α-glucoside/trehalose/maltose transporter *aglE*, also identified as AbcR1/2’s target, switches the metabolism of *Ensifer* spp. to the utilization of plant-derived dicarboxylic acids in a disaccharide-rich bulk soil to favor nodulation of pigeon pea (70). Competitive colonization of rhizosphere and other nutritionally complex environments by rhizobia presumably demands optimization of metabolic fluxes through the hierarchical utilization of available substrates. Massive but controlled silencing of metabolic mRNAs from the LsrB and RpoH1 regulons would help prevent energy-expensive uptake, catabolism, and biosynthesis of nonpriority compounds. Such a large RNA network provides additional levels of regulation relying on mRNA competition for the sRNA. First, computational predictions suggest that the binding affinity between the sRNA and the target mRNAs is a determinant of the hierarchy in the network; i.e., more extensive base pairing to the sRNA would provide priority for regulation (40). Second, competing endogenous RNAs (ceRNAs) acting as sRNA antagonists can also mediate cross-regulation of mRNAs (71). Crosstalk between ABC transporter mRNAs via a target mRNA-derived ceRNA has already been demonstrated in the GcvB regulon (72). Therefore, this is a plausible mechanism for the posttranscriptional control of AbcR1/2 levels, which likely occurs upon specific metabolic shifts or in endosymbiotic bacteria. Our MAPS data set can be further inspected to search for such AbcR1/2 sponges (73).

To conclude, our findings depict a singularly large RNA network that governs metabolic adaptations of S. meliloti during colonization of the selective alfalfa rhizosphere. Similar networks have likely diverged to help alphaproteobacteria adapt to their specific host-associated soil environments. Since AbcR1/2-targeting motifs are potentially modifiable to base pair and regulate noncognate mRNAs, this network might be rewired at different levels to engineer highly competitive biofertilizers.

## MATERIALS AND METHODS

### Bacterial strains and growth conditions.

Bacterial strains and plasmids used in this work, along with their relevant characteristics, are listed in Table S1 in the supplemental material. E. coli strains were routinely grown in lysogeny broth (LB) medium at 37°C, and rhizobia were grown in either complex tryptone-yeast (TY) medium (74) or defined mannitol-glutamate MM (75) at 30°C. To assess the stress-dependent expression of AbcR1/2, exponentially growing bacteria in MM were cultured for a further 1 h upon salt (400 mM NaCl) and heat (40°C) shocks. To test the effect of shifts in nitrogen metabolism on AbcR1/2 accumulation, the l-glutamate (6.5 mM) of the standard MM was replaced by NH_4_Cl (10 or 0.5 mM) or KNO_3_ (10 or 0.5 mM). Similarly, the impact of different carbon sources in AbcR1/2 expression was assessed in MM with ammonia (10 mM) as the nitrogen source and either mannitol (54 mM), sucrose (10 mM), glycerol (15 mM), glutamate (6.5 mM), or rhamnose (15 mM) as the carbon substrate. When required, growth media were supplemented with the appropriate antibiotic(s) (in micrograms per milliliter): streptomycin (Sm) at 480, tetracycline (Tc) at 10, erythromycin (Er) at 100, and kanamycin (Km) at 50 for E. coli and at 180 for S. meliloti.

### Oligonucleotides.

Sequences of the oligonucleotides used as probes for Northern hybridization or as amplification primers for cloning and RT-qPCR are listed in Table S2.

10.1128/mbio.03576-21.8 TABLE S2 Oligonucleotides. Download Table S2, DOCX file, 0.02 MB. Copyright © 2022 García-Tomsig et al. 2022 García-Tomsig et al. https://creativecommons.org/licenses/by/4.0/ This content is distributed under the terms of the Creative Commons Attribution 4.0 International license.

### RNA isolation and Northern blot analysis.

Total RNA was isolated from free-living bacteria cultured under the described conditions by acid phenol-chloroform extraction (76). For Northern analysis, RNA samples (15 to 20 μg) were subjected to electrophoresis on 6% polyacrylamide–7 M urea gels, blotted into nylon membranes, and probed with 5′-end-radiolabeled PbAbcR1/2 or PbAbcR2 oligonucleotides as described previously (23).

### Construction of S. meliloti mutants.

Knockout mutants were generated by deletion of the wild-type loci using the suicide plasmid pK18*mobsacB* to induce allelic replacement by double crossover (56, 77). Plasmids were mobilized to the parent strains by biparental mattings (78). SmΔ*lsrB* was generated in Sm2011 by a markerless in-frame deletion of the *lsrB* coding sequence using pK18Δ*lsrB*. To construct pK18Δ*lsrB*, 822-bp and 814-bp DNA fragments flanking the *lsrB* open reading frame (ORF) were amplified from genomic DNA with the EcoRIuplsrB/BamHIATGlsrB and BamHITGAlsrB/XbaIdownlsrB primer pairs. PCR fragments were digested with EcoRI/BamHI and BamHI/XbaI, respectively, and ligated to the pK18*mobsacB* EcoRI and XbaI restriction sites, leading to insertion of the tandem fragments via their common BamHI site. Sm2020 (triple *abcR1 abcR2 nfeR1* deletion mutant) was generated in Sm2019 (derived from Sm2011) (Table S1) by successive replacement of the three sRNA loci by a 135-bp erythromycin resistance cassette (SSDUT1) using plasmids pK18Δ*nfeR1* and pK18Δ*abcR1R2* (14, 23). Similarly, SmΔ*abcR1*, SmΔ*abcR2*, and SmΔ*abcR1R2* were generated with the parent strain Sm2B3001 (derived also from Sm2011) (Table S1) using plasmids pK18Δ*abcR1*, pK18Δ*abcR2*, and pK18Δ*abcR1R2* (15, 79). All PCR amplifications required for cloning were performed with the proofreading Phusion high-fidelity DNA polymerase (Thermo Scientific). Plasmid inserts were always checked by sequencing to confirm the absence of PCR-introduced mutations. The mutants SmΔ*lsrB*, SmΔ*abcR1*, SmΔ*abcR2*, and SmΔ*abcR1R2* were further checked by whole-genome sequencing.

### Construction of plasmids for induced AbcR1/2 expression and tagging.

For the IPTG-induced expression of wild-type and MS2 aptamer-tagged AbcR1/2, we constructed plasmids pSKiAbcR1, pSKiAbcR2, pSKiMS2AbcR1, and pSKiMS2AbcR2, which are based on the indirect *sinR-sinI* system as described previously (18). The promoter region *sinR-P_sinI_-TSS_sinI_* was PCR amplified using genomic DNA as the template with the primers sinR_NfeIF/TSS3_28bp_b_sinIR, and the MS2 aptamer was amplified from pSRKMS2 using the MS2FusTSSI/HindIIIvec primer pair (35). These two fragments overlap and were jointly used as the template for amplification with sinR_NdeIF/HindIIIvec, and the resulting PCR product was restricted with NdeI and XbaI and inserted into pSRKKm to yield pSKiMS2 (80). AbcR1 and AbcR2 were amplified from pSRKMS2AbcR1 or pSRKMS2AbcR2 (constitutively expressing tagged AbcR1 or AbcR2) using the PCR1/PCR2 primers (35). PCR products were digested with XbaI and XhoI and inserted into pSKMS2 to generate pSKiMS2AbcR1 and pSKiMS2AbcR2. Alternatively, AbcR1 and AbcR2 were amplified from pSRK-R1 or pSRK-R2 (constitutively expressing the wild-type transcripts) using the AbcR1OexfusTSSI/secSRK or AbcR2OexfusTSSI/secSRK primer pairs, respectively (15). Both forward primers contain a sequence complementary to TSS3_28bp_b_sinIR. The first PCR products were used as the template for a second PCR using the primer pair sinR_NdeIF/HindIIIvec or sinR_NdeIF/secSRK. The resulting fragments were restricted with NdeI and XbaI and inserted into pSRKKm to generate pSKiAbcR1 and pSKiAbcR2.

Replacements of specific nucleotides within aSD1/2 were performed using a two-step PCR strategy based on overlapping fragments using pSKiAbcR1 or pSKiAbcR2 as the template, as described previously (14). The first round of PCR amplifications was performed with sinR_NdeIF or secSRK (both hybridizing to all plasmid templates) and their respective primer pair carrying the desired mutations (Table S2). Each pair of complementary PCR products was used as the template in the second PCR with sinR_NdeIF/secSRK. The resulting products were digested with NdeI/XbaI and ligated to pSRKKm to yield plasmids pSKiAbcR1a, pSKAbcR1b, pSKAbcR2a, and pSKAbcR2b, which were mobilized to S. meliloti strain Sm2020 by biparental mattings.

### MS2 affinity purification coupled with RNA sequencing (MAPS).

The affinity purification assays were performed by following a previously described protocol adapted to S. meliloti (32, 33, 35, 36). Sm2020 cells carrying pSKiMS2AbcR1 or pSKiAbcR1 (control of column-binding specificity) were grown in TY and MM to exponential phase. Bacteria carrying pSKiMS2AbcR2 or the control pSKiAbcR2 were cultured in TY and MM to stationary phase or subjected to temperature and salt upshifts upon growth in TY to exponential phase. Aliquots of wild-type- and tagged-AbcR1/2-derived cultures were independently pooled, and cells equivalent to an optical density at 600 nm (OD_600_) of 200 were harvested by centrifugation (4°C) at 3,500 × *g* for 15 min after the addition of IPTG (1 mM) to induce sRNA transcription. Cells were washed with 20 mL TE buffer (pH 8), centrifuged again, resuspended in 4 mL lysis buffer (20 mM Tris-HCl, pH 8.0, 150 mM KCl, 1 mM MgCl_2_, 1 mM dithiothreitol [DTT]), and broken using a French press. Soluble cell fractions were subjected to affinity chromatography on MS2-MBP-conjugated amylose resin as described previously (35, 36). Eluted RNA was isolated by phenol-chloroform extraction followed by precipitation of the aqueous phase with 4 vol ethanol (EtOH) in the presence of 20 μg of glycogen. To monitor the procedure, RNA was obtained from 50 μl of cleared lysate, flow through, and wash fractions by acid phenol/chloroform extraction (76). These RNA preparations were probed with PbAbcR1/2 upon Northern blotting.

Strand-specific cDNA libraries from RNA fractions eluted from columns were generated and sequenced in the Illumina NextSeq Mid 150 platform. Demultiplexed sequencing reads were mapped with Bowtie2 v2.2.3 using parameters standard to the S. meliloti Sm1021 reference sequence downloaded from the RhizoGATE portal (37, 81). Uniquely mapped reads were assigned to protein-coding genes or noncoding RNAs with Rsubread 3.12 (82). Read counts for each genomic feature were normalized by coverage, and the resulting numbers of reads per kilobase per million (RPKM) were the basis for fold change calculations (83). The Integrative Genomics Viewer (IGV) software was used for data visualization (84).

### Fluorescence reporter assays.

The transcriptional fusions reporting promoter activity were generated in the promoterless vector pBB*eGFP* (14). AbcR1 (334 bp) and AbcR2 (206 bp) promoters were amplified with the primer pairs XbaIAbcR1/PC15Rv and EcoRIPC16/PC16Rv, respectively. The PCR products were digested with XbaI (P*_abcR1_*) or HindIII/XbaI (P*_abcR2_*) and cloned into pBB*eGFP* to generate pBBAbcR1::*eGFP* and pBBAbcR2::*eGFP*. Trimmed versions of both promoters (38 bp) were generated by annealing the oligonucleotides PR1_50i/PR1_50 (P*_abcR1–38_*) and PR2_58i/PR2_58 (P*_abcR2–38_*) and cloning the products into pGEM-T. P*_AbcR1–38_* and P*_AbcR2–38_* were retrieved from pGEM-T by SpeI-XbaI restriction and finally inserted in pBB*eGFP* to yield pBBAbcR1–38::*eGFP* and pBBAbcR2–38::*eGFP*.

Reporter fusions of *SMc02417* and *SMa0392* to *eGFP* were generated in plasmid pR-*eGFP* (15). For this, genomic regions of *SMc02417* and *SMa0392* from their respective transcription start sites to the 12th or 77th codons were amplified with the a0392F/a0392R and c02417F/c02417R primer pairs, respectively. The resulting PCR products were digested with BamHI/NheI and cloned into pR-*eGFP* to yield pR*SMc02417*::*eGFP* and pR*SMa0392*::*eGFP*. Compensatory nucleotide substitutions in all tested target mRNAs (i.e., *SMc03121*, *SMa0495*, *prbA*, *SMc02417*, and *SMa0392*) for regulation by the corresponding AbcR1/2 variants were introduced by a two-step PCR using the respective wild-type reporter fusion as the template. The first round of PCR amplifications was performed with PCR2 or Egfp-139_rev (both hybridizing to the plasmid templates) and their respective primer pair carrying the specific mutations (Table S2). Each pair of complementary PCR products was used as the template in the second PCR with PCR2/Egfp-139. The resulting products were digested with BamHI/NdeHI and ligated to pR-*eGFP* to generate the new set of reporters (1 and 2 variants of each wild-type reporter). All reporter plasmids were transferred by biparental conjugation to Sm2020 harboring plasmids expressing either wild-type AbcR1/2 or their a/b variants. Transconjugants for each RNA-target fusion combination were grown to exponential phase (OD_600_ of 0.2 to 0.3), divided into untreated and 0.5 mM IPTG-treated cultures, and incubated for 24 h. OD_600_ and fluorescence (excitation, 485 nm; emission, 520 nm) were measured in a Thermo Scientific Varioskan LUX multimode microplate reader. Fluorescence values were normalized to the culture OD_600_.

### Electrophoretic mobility shift assays (EMSA) with LsrB.

The LsrB coding sequence was PCR amplified from genomic DNA using the primers LsrB_Fw_ndeI/LsrB_Rv_BamHI and cloned into the vector pET-16b (Novagen) between the NdeI/BamHI restriction enzymes sites, yielding p16LsrB encoding a His-tagged LsrB. Recombinant LsrB was produced and purified as described previously (35). The EcoRIPC16/PC16Rv and XbaIAbcR1/PC15Rv primer pairs were used to amplify P*_abcR1_* (334 bp) and P*_abcR2_* (206 bp), respectively, which were further purified from agarose gels with the GFX PCR DNA and Gel Band purification kits (GE Healthcare). Binding reactions were performed with 100 nM radiolabeled probes in the absence or presence (1 μM) of purified LsrB and then subjected to electrophoresis and analyzed with the Personal FX equipment and Quantity One software (Bio-Rad) as described previously (85).

### RT-qPCR.

RNA samples obtained as described previously were further cleaned up with the RNeasy minikit (Qiagen) following DNase digestion. cDNA was synthesized with the qScript cDNA synthesis kit (Quantabio). RT-qPCR was carried out in a QuantStudio 3 system (Thermo Fisher Scientific) using the Takyon low Rox SYBR 2× master mix blue dTTP (Eurogentec). The ratios of transcript abundance were calculated as the ΔΔ*CT* (where *CT* is threshold cycle) mean average of results from three replicates using three independent RNA extracts, where the ΔΔ*CT* represents the level of gene expression in the IPTG-induced strain relative to that in the untreated control strain. The seemingly constitutive gene *SMc01852*, encoding a phosphofructokinase, was used to normalize gene expression (86). Control reactions without reverse transcriptase (–RT) in the RNA samples were simultaneously performed to confirm an absence of DNA contamination.

### Root colonization assays.

Alfalfa (*Medicago sativa* L. “Aragón”) plants were sterilized, germinated, and grown in hydroponic cultures under axenic conditions as described previously (87). Root colonization by bacteria was assessed by counting CFU. Rhizobial strains were grown on TY plates at 30°C for 2 days, and the cell mass was resuspended in TY broth to an OD_600_ of 0.5 and then diluted 100-fold in sterile water to prepare an inoculum of approximately 10^6^ bacterial cells/plant. At defined times, 15 roots inoculated with each rhizobial strain were washed 3 times with 20 mL sterile water to remove the loosely attached bacteria, and the roots were weighed in groups of five placed into 2-mL Eppendorf tubes. Then, 1 mL of sterile TE buffer was added to each tube, and the attached cells were released by two sonication pulses of 1 min each in an Ultrasons sonicator bath with a pause time of 1 min between the pulses. Cells were subsequently quantified by counting CFU (normalized to grams of root). Experiments were conducted in triplicate for each tested strain.

### Computational methods.

Promoter sequence alignments were generated with ClustalW implemented in BioEdit (88), and searches for conserved motifs were done with the MEME algorithm (89; http://meme-suite.org/index.html). The logo of the motif consensus sequence was generated at http://weblogo.berkeley.edu/logo.cgi. Venn diagrams were generated with the Venny 2.0 tool (https://bioinfogp.cnb.csic.es/tools/venny/index2.0.2.html). CopraRNA (v 2.1.2) and IntaRNA (v 3.2.0; http://rna.informatik.uni-freiburg.de/) were used to predict sRNA-mRNA base-pairing interactions (52, 90).

FBA simulations were performed in MATLAB R2019a (MathWorks) using SBMLToolbox version 4.1.0 (91), libSBML version 5.17 (92), scripts from the COBRA Toolbox commit 6a99a1e (93), and the iLOG CPLEX Studio 12.9.0 solver (ibm.com). All analyses were performed on the S. meliloti metabolic model iGD1348 (39). For each nutrient condition that was tested, the maximal growth rate and the overall metabolic flux rates of iGD1348 were determined using the “optimizeCbModel” and “pFBA” functions of the COBRA Toolbox. Then, each gene belonging to the AbcR1/2 targetome was either individually or simultaneously deleted, and the maximal growth rate and the overall metabolic flux rates were determined. AbcR1/2 were predicted to regulate transport/metabolism during growth with a carbon source when deletion of any individual gene or the entire targetome resulted in a growth rate of <90% of that of the wild-type model or an overall flux rate of >110% that of the wild-type model. Analyses in simulated bulk soil and rhizosphere conditions were performed using previously defined nutritional conditions (94). Code to repeat the analyses is provided as Text S1.

10.1128/mbio.03576-21.1 TEXT S1 Code for iGD1348-based analysis of the AbcR1/2 targetomes. Download Text S1, TXT file, 0.03 MB. Copyright © 2022 García-Tomsig et al. 2022 García-Tomsig et al. https://creativecommons.org/licenses/by/4.0/ This content is distributed under the terms of the Creative Commons Attribution 4.0 International license.

The 23 complete S. meliloti genomes present in the ftp NCBI folder on 17 February 2021 were downloaded, and the pangenome was computed with the Pan/Core-Genome and the Gene Phyloprofile tools of the MicroScope platform (95), with thresholds of 80 % amino acid identity and 80 % alignment coverage, as previously reported (96). The list of AbcR1/2 target genes was used as a query to investigate the pattern of expression in S. meliloti transcriptomic data from cultures treated with alfalfa root exudates and luteolin (69) as a proxy of rhizospheric conditions.

### Data availability.

Raw RNA-seq data can be accessed at the SRA database (BioProject ID PRJNA735891).
