# Supplementary figures and images for: Pervasive RNA Regulation of Metabolism Enhances the Root Colonization Ability of Nitrogen-Fixing Symbiotic α-Rhizobia
Source: mBio. 2022 Feb 15;13(1):e03576-21. doi: 10.1128/mbio.03576-21 (PMC8844928; doi:10.1128/mbio.03576-21)

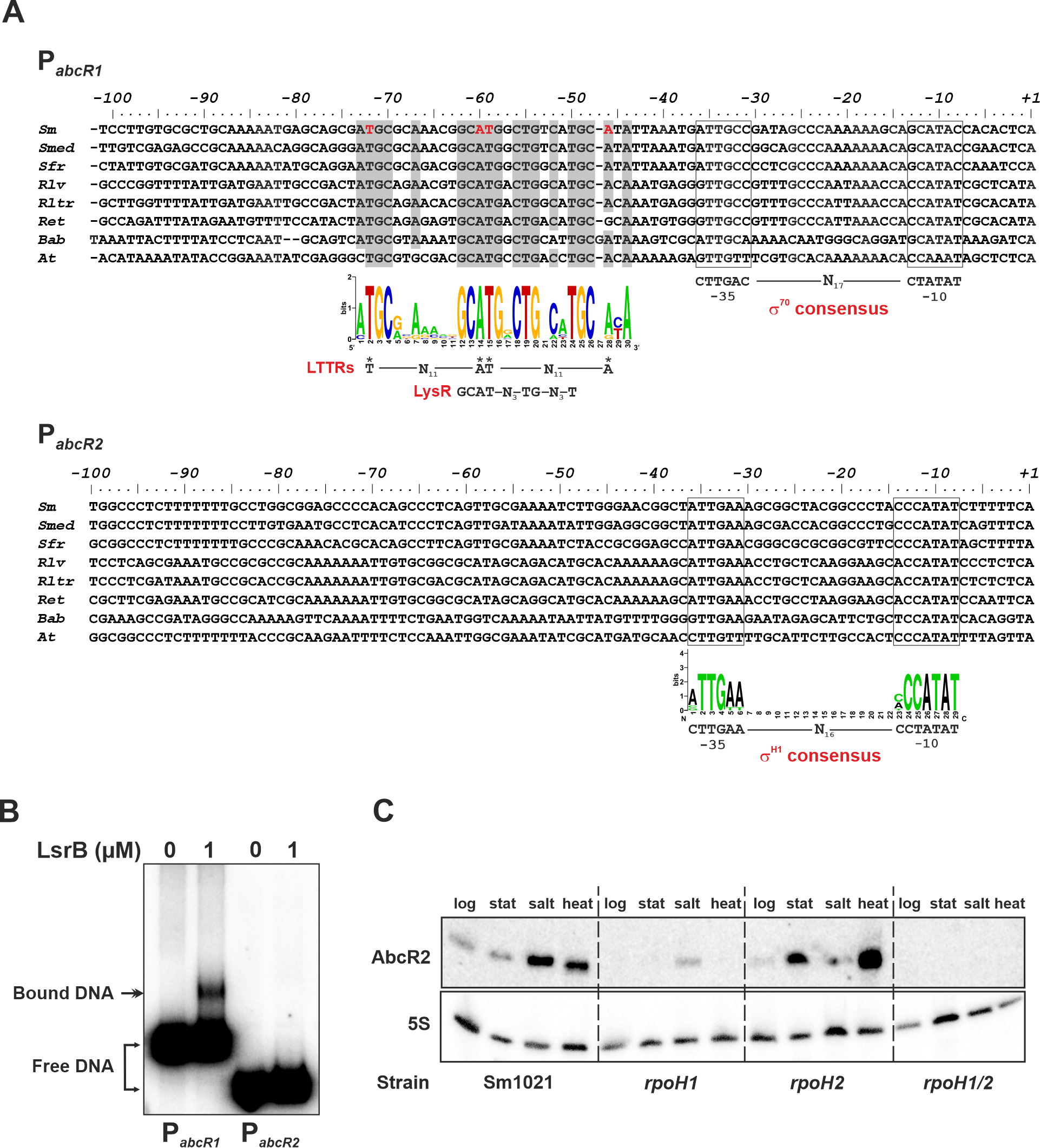

Supplement: FIG S1 [file mbio.03576-21-sf001.tif]

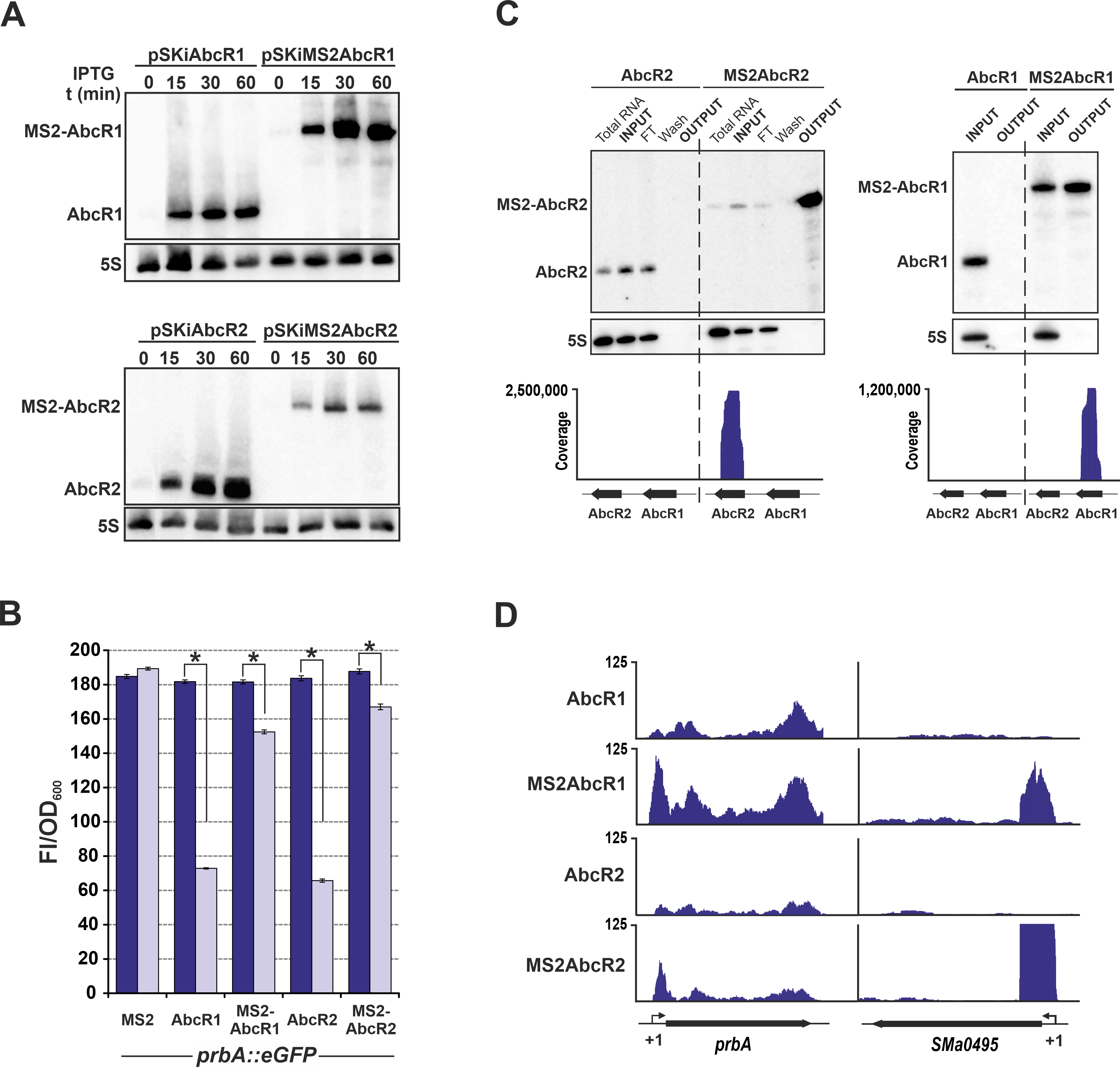

Supplement: FIG S2 [file mbio.03576-21-sf002.tif]

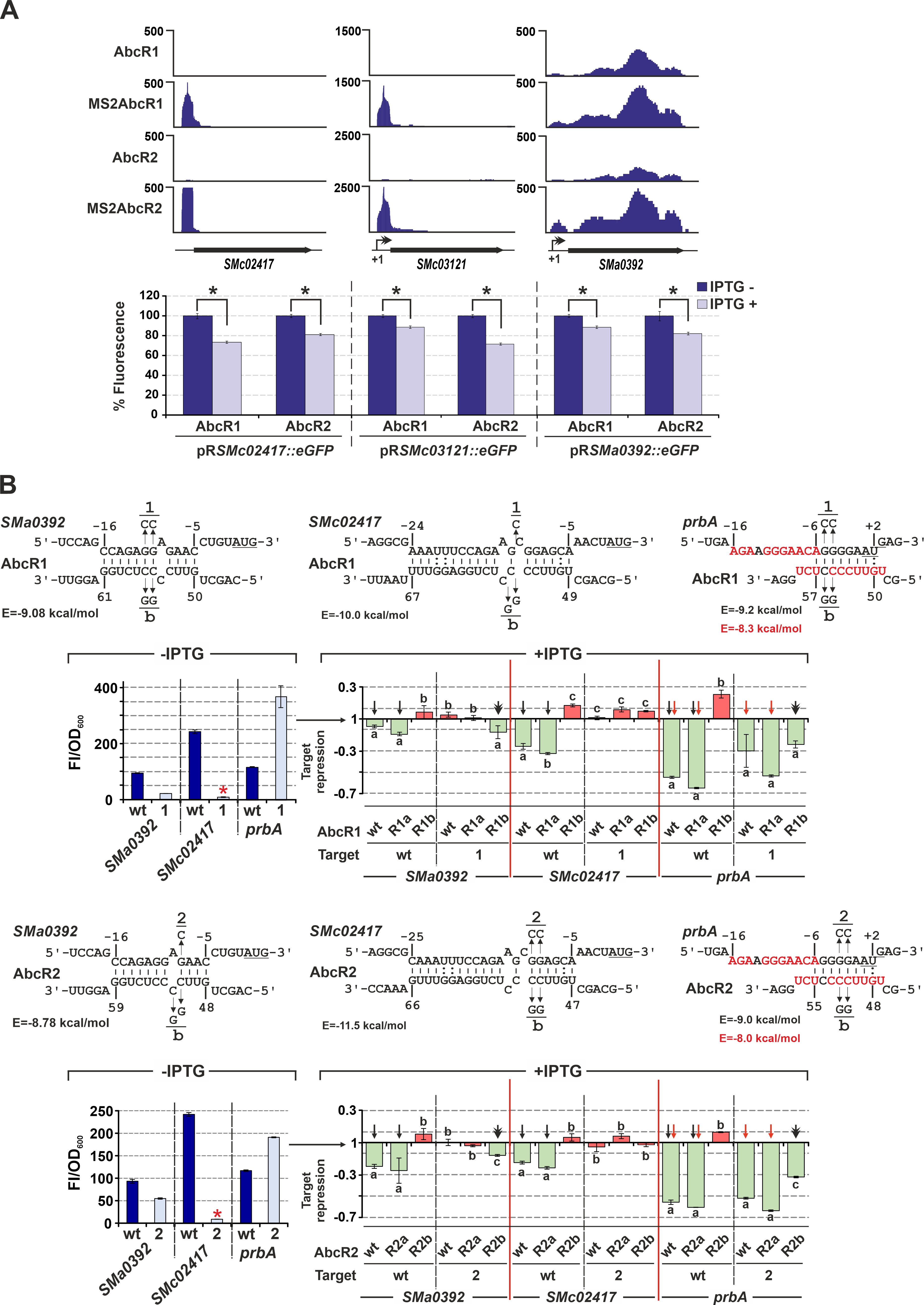

Supplement: FIG S3 [file mbio.03576-21-sf003.tif]

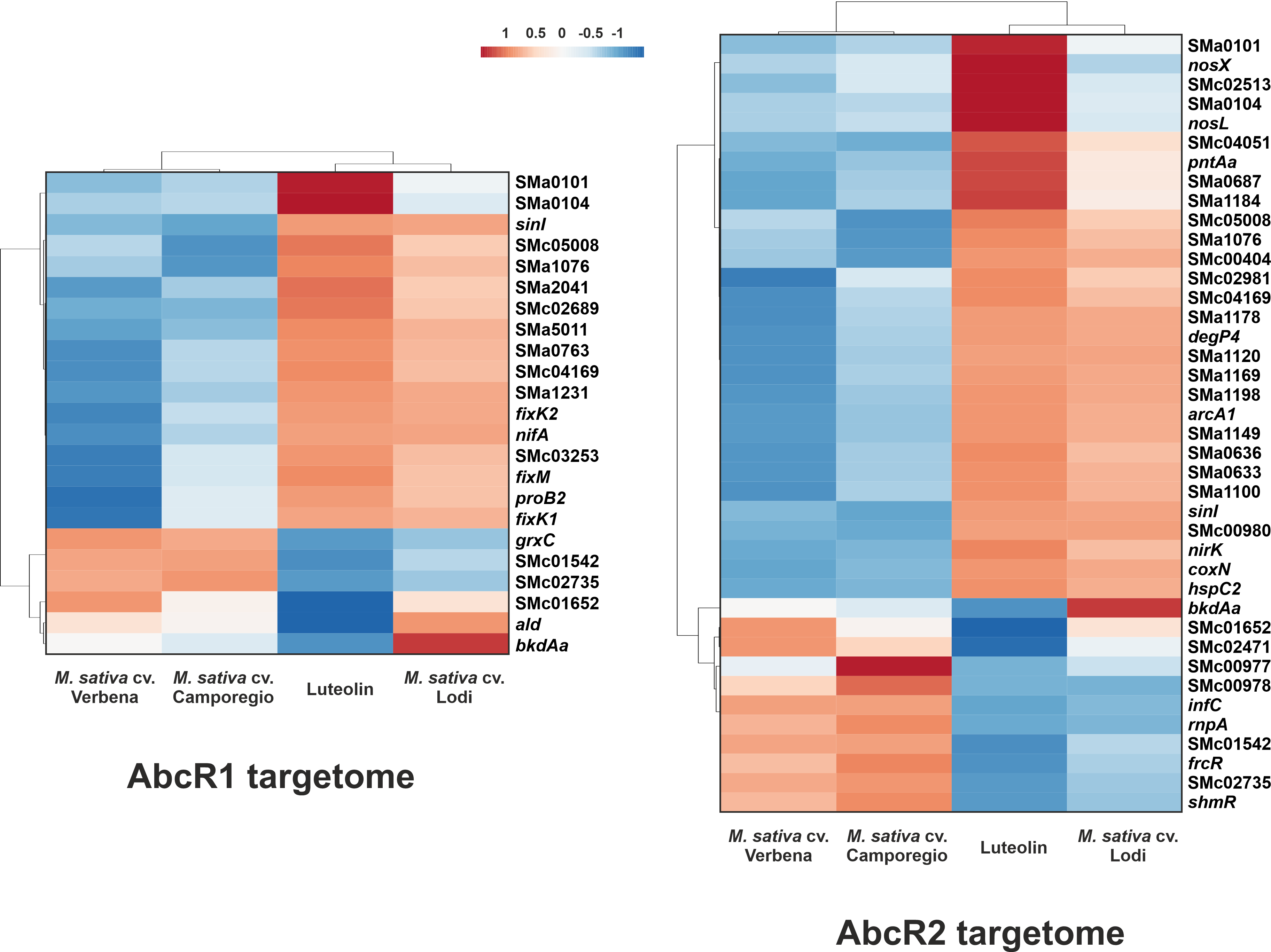

Supplement: FIG S4 [file mbio.03576-21-sf004.tif]

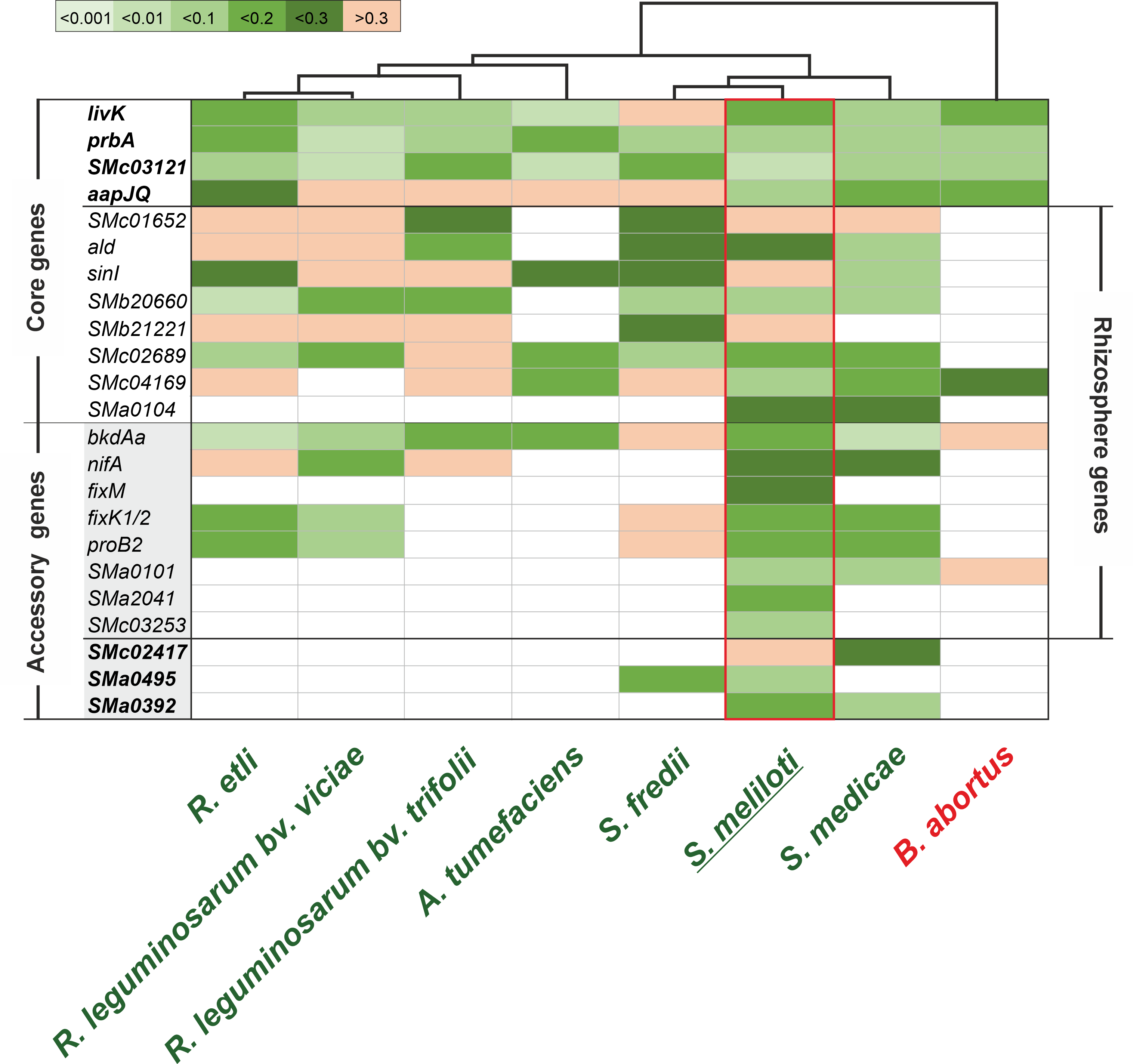

Supplement: FIG S5 [file mbio.03576-21-sf005.tif]
