# Supplementary material for: Pervasive RNA Regulation of Metabolism Enhances the Root Colonization Ability of Nitrogen-Fixing Symbiotic α-Rhizobia
Source: mBio. 2022 Feb 15;13(1):e03576-21. doi: 10.1128/mbio.03576-21 (PMC8844928; doi:10.1128/mbio.03576-21)
Supplement: TABLE S1 [file mbio.03576-21-st001.docx]

**Table S1.** Bacterial strains and plasmids used in this work

| **Strain/Plasmid** | **Relevant characteristics** | **Reference/Source** |
| --- | --- | --- |
| ***BACTERIA*** |  |  |
| ***S. meliloti*** |  |  |
| **Sm2011** | SU47 derivative; Sm^r^ | J. Denarie (C.N.R.A., Versailles) |
| **SmΔ*lsrB*** | Sm2011 ∆*lsrB* derivative; Sm^r^ | This work |
| **Sm1021** | SU47 derivative; Sm^r^ | (1) |
| **VO3128** | Sm1021 *rpoH1::aadA* derivative; Sm^r^, Sp^r^ | (2) |
| **AB3** | Sm1021 *rpoH2:: aacCI* derivative; Sm^r^, Gm^r^ | (2) |
| **AB9** | Sm1021 *rpoH1::aadA rpoH2::aacCI* derivative; Sm^r^, Gm^r^, Sp^r^ | (2) |
| **Sm2B3001** | Sm2011 *expR^+^* derivative; Nal^r^, Sm^r^ | (3) |
| **Sm2019** | Sm2B3001 derivative; markerless Δ*expR,* ∆*sinRI*; Nal^r^, Sm^r^ | M. McIntosh (SYNMIKRO, Marburg) |
| **Sm2020** | Sm2019 ∆*abcR1*/*abcR2*/*nfeR1* derivative | This work |
| **SmΔ*abcR1*** | Sm2B3001 ∆*abcR1* derivative | This work |
| **SmΔ*abcR2*** | Sm2B3001 ∆*abcR2* derivative | This work |
| **SmΔ*abcR1/2*** | Sm2B3001 ∆*abcR1/2* derivative | This work |
| ***E. coli*** |  |  |
| **DH5α** | F^–^*endA1glnV44thi-1recA1relA1gyrA96deoRnupGpurB20* φ80d*lacZ*ΔM15 Δ(*lacZYA-argF*)U169, hsdR17(*r_K_*^–^*m_K_*^+^), λ^–^ | Bethesda Research Lab |
| **S17-1** | *recA pro hsdR RP4-2-Tc::Mu-Km::Tn7* | (4) |
| **BL21(DE3)** | *E. coli str. B F– ompT gal dcmlonhsdS_B_(r_B_^–^m_B_^–^) λ(DE3 [lacI lacUV5-T7p07 ind1 sam7 nin5]) [malB^+^]_K-12_(λ^S^)* | Novagen |
| ***PLASMIDS*** |  |  |
| **pK18*mobsacB*** | Suicide plasmid in *S. meliloti*, *sacB*, *oriV*, Km^r^ | (5) |
| **pK18Δ*nfeR1*** | Suicide plasmid for *nfeR1* deletion; Er^r^, Km^r^ | (6) |
| **pK18Δ*abcR1*** | Suicide plasmid for *abcR1* deletion; Er^r^, Km^r^ | (7) |
| **pK18Δ*abcR2*** | Suicide plasmid for *abcR2* deletion; Er^r^, Km^r^ | (7) |
| **pK18Δ*abcR1R2*** | Suicide plasmid for *abcR1/abcR2* deletion; Er^r^, Km^r^ | (7) |
| **pK18Δ*lsrB*** | Suicide plasmid for *lsrB* deletion; Km^r^ | This work |
| **pSRKKm** | pBBR1MCS-2 derivative with a P*_lac_* promoter, *lacIq, lacZa*^+^, Km^r^ | (8) |
| **pSRK-MS2T** | pSRK derivative harboring the 43-nt MS2 aptamer sequence | (9) |
| **pSRK-R1** | pSRK derivative constitutively expressing AbcR1 | (7) |
| **pSRK-R2** | pSRK derivative constitutively expressing AbcR2 | (7) |
| **pSKiAbcR1** | pSRKKm carrying the AbcR1 coding sequence fused to *sinR*-P*_sinI_* | This work |
| **pSKiAbcR2** | pSRKKm carrying the AbcR2 coding sequence fused to *sinR*-P*_sinI_* | This work |
| **pSKMS2** | pSRKKm carrying the aptamer MS2 coding sequence fused to *sinR*-P*_sinI_* | This work |
| **pSKiMS2AbcR1** | pSKMS2 derivative expressing MS2-  AbcR1 | This work |
| **pSKiMS2AbcR2** | pSKMS2 derivative expressing MS2-  AbcR2 | This work |
| **pSKiAbcR1a** | pSKiAbcR1 derivative expressing AbcR1 carrying G26G27 substitutions | This work |
| **pSKiAbcR1b** | pSKiAbcR1 derivative expressing AbcR1 carrying G55G56 substitutions | This work |
| **pSKiAbcR2a** | pSKiAbcR2 derivative expressing AbcR2 carrying G28G29 substitutions | This work |
| **pSKiAbcR2b** | pSKiAbcR2 derivative expressing AbcR2 carrying G51G52 substitutions | This work |
| **pBB-*eGFP*** | pBBR1MCS-2 derivative for generation of promoter eGFP fusions; Km^r^ | (6) |
| **pBBAbcR1-38*::eGFP*** | pBBR1MCS-2 derivative expressing a transcriptional fusion of a truncated *abcR1* promoter (38-bp) to *egfp*; Km^r^ | This work |
| **pBBAbcR1*::eGFP*** | pBBR1MCS-2 derivative expressing a transcriptional fusion of the full-length *abcR1* promoter (334 bp) to *egfp*; Km^r^ | This work |
| **pBBAbcR2-38*::eGFP*** | pBBR1MCS-2 derivative expressing a transcriptional fusion of a truncated *abcR2* promoter (38-bp) to *egfp*; Km^r^ | This work |
| **pBBAbcR2*::eGFP*** | pBBR1MCS-2 derivative expressing a transcriptional fusion of the full-lenght *abcR2* promoter (206 bp) to *egfp*; Km^r^ | This work |
| **pR-*eGFP*** | Vector for generation of target mRNA-*egfp* translational fusions | (7) |
| **pR*SMc03121::eGFP*** | *SMc03121::eGFP* translational fusion (-156/+36 relative to *SMc03121* AUG) | (6) |
| **pR*SMa0495::eGFP*** | *SMa0495::eGFP* translational fusion (-78/+54 relative to *SMa0495* AUG) | (10) |
| **pR*prbA::eGFP*** | *prbA::egfp* translational fusion (-157/+48 relative to *prbA* AUG) | (10) |
| **pR*SMc02417::eGFP*** | *SMc02417::egfp* translational fusion (-372/+78 relative to *SMc02417* AUG) | This work |
| **pR*SMa0392::eGFP*** | *SMa0392::eGFP* translational fusion (-168/+231 relative to *SMa0392* AUG) | This work |
| **pET16b** | Bacterial vector for inducible expression of N-terminally 10xHis-tagged proteins with a Factor Xa site | Novagen |
| **p16*lsrB*** | pET16b derivate carrying N-terminally 10xHis-tagged LsrB | This work |

**Table S1 References**

1. Meade HM, Long SR, Ruvkun GB, Brown SE, Ausubel FM. 1982. Physical and genetic characterization of symbiotic and auxotrophic mutants of *Rhizobium meliloti* induced by transposon Tn*5* mutagenesis. *J Bacteriol* 149:114–122.

2. Barnett MJ, Bittner AN, Toman CJ, Oke V, Long SR. 2012. Dual RpoH sigma factors and transcriptional plasticity in a symbiotic bacterium. *J Bacteriol* 194:4983–4994.

3. Bahlawane C, McIntosh M, Krol E, Becker A. 2008. *Sinorhizobium meliloti* regulator MucR couples exopolysaccharide synthesis and motility. *Mol Plant Microbe Interact* 21:1498–1509.

4. Simon R, Priefer U, Puhler A. 1983. A broad host range mobilization system for in vivo genetic engineering: transposon mutagenesis in gram negative bacteria. *Nat Biotech* 1:784–791.

5. Schafer A, Tauch A, Jager W, Kalinowski J, Thierbach G, Puhler A. 1994. Small mobilizable multi-purpose cloning vectors derived from the *Escherichia coli* plasmids pK18 and pK19: selection of defined deletions in the chromosome of *Corynebacterium glutamicum*. *Gene* 145:69–73.

6. Robledo M, Peregrina A, Millán V, García‐Tomsig NI, Torres‐Quesada O, Mateos PF, Becker A, Jiménez‐Zurdo JI. 2017. A conserved α‐proteobacterial small RNA contributes to osmoadaptation and symbiotic efficiency of rhizobia on legume roots. *Environ Microbiol* 19:2661–2680.

7. Torres-Quesada O, Millán V, Nisa-Martínez R, Bardou F, Crespi M, Toro N, Jiménez-Zurdo JI. 2013. Independent activity of the homologous small regulatory RNAs AbcR1 and AbcR2 in the legume symbiont *Sinorhizobium meliloti*. *PLoS One* 8:1080–1091.

8. Khan SR, Gaines J, Roop RM, Farrand SK. 2008. Broad-host-range expression vectors with tightly regulated promoters and their use to examine the influence of TraR and TraM expression on Ti plasmid quorum sensing. *Appl Environ Microbiol* 74:5053–5062.

9. Robledo M, García-Tomsig NI, Matia-González AM, García-Rodríguez FM, Jiménez-Zurdo JI. 2021. Synthetase of the methyl donor S-adenosylmethionine from nitrogen-fixing α-rhizobia can bind functionally diverse RNA species. *RNA Biol* 18:1111–1123.

10. Torres-Quesada O, Reinkensmeier J, Schluẗer J-P, Robledo M, Peregrina A, Giegerich R, Toro N, Becker A, Jiménez-Zurdo JI. 2014. Genome-wide profiling of Hfq-binding RNAs uncovers extensive post-transcriptional rewiring of major stress response and symbiotic regulons in *Sinorhizobium meliloti*. *RNA Biol* 11:563–579.
