# Supplementary material for: Pervasive RNA Regulation of Metabolism Enhances the Root Colonization Ability of Nitrogen-Fixing Symbiotic α-Rhizobia
Source: mBio. 2022 Feb 15;13(1):e03576-21. doi: 10.1128/mbio.03576-21 (PMC8844928; doi:10.1128/mbio.03576-21)
Supplement: TABLE S2 [file mbio.03576-21-st002.docx]

**Table S2.** Oligonucleotides used in this work.

| Oligonucleotides | 5’-sequence-3’ | Use |
| --- | --- | --- |
| PbAbcR2 | GAGGAGAAAGCCGCTAGATGCACCA | Northern blot probing |
| PbAbcR1/2 | AACCTCCAGAGGGGAACAGCTGCTG |  |
| Pb5S | TACTCTCCCGCGTCTTAAGACGAA |  |
| EcoR1uplsrB | CCAGGAATTCGGATCAAGACGGATAGCG | Construction and PCR verification of SmΔ*lsrB* mutant |
| BamHIATGlsrB | GAGAGGATCCCCATAAAGGCTCAGCCGGA |  |
| BamHITGAlsrB | CTCTGGATCCACTTCTGACGATCCGTTC |  |
| XbaIdownlsrB | CGACTCTAGAACTGCCCTGCGCAGACG |  |
| checklsrBin | GAACATGCATCGGTCGTCTC |  |
| checklsrBout | CGGTAGAAGACCCATTGGC |  |
| sinR_NdeIF | GCCACATATGGCTAATCAACAGGCTGTC | Construction of pSKiAbcR1, pSKiAbcR2, pSKiMS2AbcR1 and pSKiMS2AbcR2 |
| TSS3_28bp_b_sinIR | GTAGCGATGCTGTCAGGCTC |  |
| MS2fusTSSI | GAGCCTGACAGCATCGCTACCGTACACCATCAGGGTAC |  |
| HindIIIVec | CGAGGTCGACGGTATCGATAAGCTTCGCC |  |
| PCR1 | CGGGCCTCTTCGCTATT |  |
| PCR2 | TTAGCTCACTCATTAGG |  |
| AbcR1OexfusTSSI | GAGCCTGACAGCATCGCTACAGCTGATGCATCTTTGGTG |  |
| AbcR2OexfusTSSI | GAGCCTGACAGCATCGCTACAGCTGGTGCATCTAGCGG |  |
| SecSRK | TTCCATTCGCCATTCAGGCT |  |
| R1G26G27_R | CCAGAAGCCACCAAAGATGC | Construction of pSKiAbcR1a combined with sinR_NdeIF (R) and SecSRK (F) |
| R1G26G27_F | GCATCTTTGGTGGCTTCTGGTCCCAGTGCCACCGCAGC |  |
| R1G55G56_R | CCGGAACAGCTGCTGCGGTGG | Construction of pSKiAbcR1b combined with sinR_NdeIF (R) and SecSRK (F) |
| R1G55G56_F | CCACCGCAGCAGCTGTTCCGGTCTGGAGGTTTTAATTACC |  |
| R2G28G29_R | CCAGGAGAAAGCCGCTAGATG | Construction of pSKiAbcR2a combined with sinR_NdeIF (R) and SecSRK (F) |
| R2G28G29_F | CATCTAGCGGCTTTCTCCTGGCCAGCCGCTGCAGCAGCTGT |  |
| R2G51G52_R | CCAACAGCTGCTGCAGCGGC | Construction of pSKiAbcR2b combined with sinR_NdeIF (R) and SecSRK (F) |
| R2G51G52_F | GCCGCTGCAGCAGCTGTTGGCCTCTGGAGGTTTGAAACCTT |  |
| XbaIAbcR1 | GCCGTCTAGAGCTGATGCATCTTTGGTGGC | P*_abcR1_* amplification |
| PC15Rv | TCTAGAAGCCGCTAGATGCACCTGCT |  |
| EcoRIPC16 | CGTCGAATTCTGCCGATAAGCGCCGATA | P*_abcR2_* amplification |
| PC16Rv | TCTAGAGATGCATCAGCTGAGTGTGG |  |
| PR1_50i | CTAGAGATGCATCAGCTGAGTGTGGTATGCTGCTTTTTTGGGCTATCGGCAATCAA | Generation of P*_abcR1-38_* by annealing |
| PR1_50 | CTAGTTGATTGCCGATAGCCCAAAAAAGCAGCATACCACACTCAGCTGATGCATCT |  |
| PR2_58i | CTAGAAGCCGCTAGATGCACCAGCTGAAAAAGATATGGGTAGGGCCGTAGCCGCTTTCAATAGA | Generation of P*_abcR2-38_* by annealing |
| PR2_58 | CTAGTCTATTGAAAGCGGCTACGGCCCTACCCATATCTTTTTCAGCTGGTGCATCTAGCGGCTT |  |
| SMc02417_F | GCTAGCATCGTTTATGGATTCCATCC | Amplification of the *SMc02417* 5’-region fused to *eGFP* |
| SMc02417_R | GGATCCCTCAAGAGCACGCAATTTCG |  |
| a0392_F | GGATCCATCCGGGTTCCGGATCTG | Amplification of the *SMa0392* 5’-region fused to *eGFP* |
| a0392_R | GCTAGCGACGGCAATCCCGGTCAT |  |
| Egfp-139_rev | GATGAACTTCAGGGTCAGCTTG | Cloning in pBB-*eGFP* |
| a0392R1R | GGTCTGGCTGGACCTTCTGG | Compensatory mutations in *SMa0392* 5’-region for targeting with AbcR1b or AbcR2b |
| a0392R1F | CCAGAAGGTCCAGCCAGACCAGAACCTGTAATGTCGC |  |
| a0392R2R | GTCCTCTGGCTGGACCTTC |  |
| a0392R2F | GAAGGTCCAGCCAGAGGACAACCTGTAATGTCGC |  |
| a0495R1R | GGTCTCGTTTTTTCTGGTACC | Compensatory mutations in *SMa0495* 5’-region for targeting with AbcR1b or AbcR2b |
| a0495R1F | GGTACCAGAAAAAACGAGACCGGAATGAACGCAATGAAAAAC |  |
| a0495R2R | GGCCTCTCGTTTTTTCTGGTACC |  |
| a0495R2F | GGTACCAGAAAAAACGAGAGGCCAATGAACGCAATGAAAAAC |  |
| prbAR1R | GGTGTTCCCTTCTTCAGCCG | Compensatory mutations in *prbA* 5’-region for targeting with AbcR1b or AbcR2b |
| prbAR1F | CGGCTGAAGAAGGGAACACCGGAATGAGCGATTACAAAG |  |
| prbAR2R | GGCCTGTTCCCTTCTTCAGC |  |
| prbAR2F | GCTGAAGAAGGGAACAGGCCAATGAGCGATTACAAAGAC |  |
| c02417R1R | GTTCTGGAAATTTGCGCCTC | Compensatory mutations in *SMc02417* 5’-region for targeting with AbcR1b or AbcR2b |
| c02417R1F | GAGGCGCAAATTTCCAGAACCGGAGCAAACTTATGATG |  |
| c02417R2R | GGGCTTCTGGAAATTTGCGCC |  |
| c02417R2F | GGCGCAAATTTCCAGAAGCCCAGCAAACTTATGATGAAG |  |
| c03121R1R | GGTCCCAAAGGTTCTATGTTC | Compensatory mutations in *SMc03121* 5’-region for targeting with AbcR1a or AbcR2a |
| c03121R1F | GAACATAGAACCTTTGGGACCAGGACAAATGCACAAGAAAC |  |
| c03121R2R | GGCAAAGGTTCTATGTTCTAAAG |  |
| c03121R2F | CTTTAGAACATAGAACCTTTGCCAGGAGGACAAATGCACAAG |  |
| LsrB_Fw_NdeI | ATATCATATGGGGGATTCTATGTCGCT | Amplification of the LsrB coding sequence |
| LsrB_Rv_BamHI | ATGGATCCTTATCAGAAGTTCCAGTTTCTCG |  |
| aapQ594F | CGCCGCAAGTGTGTTCTTTG | qRT-PCR of *aapQ* |
| aapQ712R | GAAATGTGACCAGCGGCAGA |  |
| Smc01852F | TCACCAACACTGCCGACTGC |  |
| Smc01852R | TCGTGTGCAGGATGCTGATG |  |

Restriction sites are underlined.
